# A Chest‐Laminated Ultrathin and Stretchable E‐Tattoo for the Measurement of Electrocardiogram, Seismocardiogram, and Cardiac Time Intervals

**DOI:** 10.1002/advs.201900290

**Published:** 2019-05-21

**Authors:** Taewoo Ha, Jason Tran, Siyi Liu, Hongwoo Jang, Hyoyoung Jeong, Ruchika Mitbander, Heeyong Huh, Yitao Qiu, Jason Duong, Rebecca L. Wang, Pulin Wang, Animesh Tandon, Jayant Sirohi, Nanshu Lu

**Affiliations:** ^1^ Department of Electrical and Computer Engineering University of Texas at Austin TX 78712 USA; ^2^ Department of Aerospace Engineering and Engineering Mechanics University of Texas at Austin TX 78712 USA; ^3^ Texas Materials Institute University of Texas at Austin TX 78712 USA; ^4^ Department of Biomedical Engineering University of Texas at Austin TX 78712 USA; ^5^ Department of Mechanical Engineering University of Texas at Austin TX 78712 USA; ^6^ Departments of Pediatrics, Radiology, and Biomedical Engineering Division of Cardiology University of Texas Southwestern Medical School Children's Medical Center Dallas TX 75235 USA

**Keywords:** blood pressure, cardiac time intervals, digital image correlation, epidermal electronics, e‐tattoos

## Abstract

Seismocardiography (SCG) is a measure of chest vibration associated with heartbeats. While skin soft electronic tattoos (e‐tattoos) have been widely reported for electrocardiogram (ECG) sensing, wearable SCG sensors are still based on either rigid accelerometers or non‐stretchable piezoelectric membranes. This work reports an ultrathin and stretchable SCG sensing e‐tattoo based on the filamentary serpentine mesh of 28‐µm‐thick piezoelectric polymer, polyvinylidene fluoride (PVDF). 3D digital image correlation (DIC) is used to map chest vibration to identify the best location to mount the e‐tattoo and to investigate the effects of substrate stiffness. As piezoelectric sensors easily suffer from motion artifacts, motion artifacts are effectively reduced by performing subtraction between a pair of identical SCG tattoos placed adjacent to each other. Integrating the soft SCG sensor with a pair of soft gold electrodes on a single e‐tattoo platform forms a soft electro‐mechano‐acoustic cardiovascular (EMAC) sensing tattoo, which can perform synchronous ECG and SCG measurements and extract various cardiac time intervals including systolic time interval (STI). Using the EMAC tattoo, strong correlations between STI and the systolic/diastolic blood pressures, are found, which may provide a simple way to estimate blood pressure continuously and noninvasively using one chest‐mounted e‐tattoo.

## 1 Introduction

Cardiovascular diseases (CVD) are the leading cause of death in the United States and cost the nation hundreds of billions of dollars each year.^1^ As a result, wearable devices are being developed to perform continuous cardiovascular monitoring for telemedicine and outpatients.^2^ Among all the cardiovascular signals, the best known is electrocardiogram (ECG), which indicates the electrical activity of the heart. Wearable devices such as Holter monitors have been developed to track ECG continuously.^3^ While ECG reflects myocardial conduction, myocardial contraction is characterized by mechano‐acoustic signals.^4^ These signals provide important insights into cardiovascular health that complement those inferred from ECG. Typical mechanoacoustic signals of the heart include phonocardiogram (PCG, sounds made by the heart),^5^ seismocardiogram (SCG, local vibrations of the chest wall caused by a heartbeat),^6^ and ballistocardiogram (BCG, a whole‐body movement generated by the sudden ejection of blood into the vasculature).^6^ PCG is measured by stethoscopes. SCG can be recorded using a digital accelerometer attached to the chest.^7^, ^8^ BCG should be measured with a swing bed or a force sensor placed on a weighing scale.^6^ These mechano‐acoustic signals, although measured differently, all emerge from the mechanical activities of the heart.

Since epidermal electronics has been introduced,^9^ many advances have been made in soft ECG electrodes.^10^, ^11^ In contrast, there has been little progress in the development of soft SCG sensors. Although SCG can be measured by mounting commercial accelerometers on the human chest,^6^ the thickness and rigidity of conventional accelerometers make them uncomfortable to wear and susceptible to the global inertial motion of the body.^7^ Physical strapping or taping is also required to reduce the acoustic mismatch caused by the air gap between the rigid accelerometer and the skin.^7^ To overcome this issue, Liu et al. proposed a soft and stretchable circuit using serpentine interconnects and elastomeric encapsulation to replace a rigid PCB.^7^ While the soft circuit is already a big improvement over a rigid PCB, the accelerometer mounted on the soft circuit is still a 2‐mm‐thick rigid chip, and the fabrication relies on expensive photolithography and micro‐transfer processes.

Other than conventional accelerometers, piezoelectric transducers can also detect SCG because they are able to convert mechanical motion to electric charges.^12^ Passive sensing is a unique advantage of piezoelectric transducers since they can generate electric charges without external power supply. In fact, an unpatterned electromechanical film (EMFi, EMFIT Ltd, Finland) has been taped on the chest for SCG measurement.^13^ However, the in‐plane elastic stiffness of EMFi is 0.5–1 GPa^14^ which is orders of magnitude higher than that of human skin (130 kPa to 20 MPa^15^). Although there have been stretchable piezoelectric devices based on patterned inorganic piezoelectric materials such as lead zirconate titanate (PZT) or zinc oxide (ZnO),^16^ they are intrinsically brittle materials, expensive to produce, and even contain hazardous substance such as lead. In contrast, polyvinylidene fluoride (PVDF) is a commercially available piezoelectric polymer that is mechanically robust and biocompatible. Its Young's modulus is 3.6 GPa hence unpatterned PVDF is not stretchable either.^17^, ^18^ Stretchable electromechanical sensors or energy harvesters based on patterned PVDF have emerged in recent years. For example, micropatterned PVDF membrane,^19^ ribbon‐like PVDF embedded in Ecoflex,^20^ and PVDF islands interconnected by serpentine metal wires^21^ have shown around a 30% stretchability, but their thinness and softness have not been explicitly determined. Several wearable sensors and energy harvesters have employed electrospun PVDF fibers, but electrodes could only be placed at the ends of the fiber bundles instead of along the full length of the fiber,^22^ which compromises the electrical output and, subsequently, the sensitivity. So far, a low‐cost, ultrathin, and skin‐soft 28‐µm‐thick SCG sensor based on PVDF is still lacking.

Here, we propose to pattern commercially available metalized PVDF sheets into a filamentary serpentine (FS) network as an ultrathin and stretchable mechano‐acoustic sensor, with special emphasis on its thickness, softness, stretchability, manufacturability, and sensitivity. The commercial PVDF sheet is 28 µm thick and comes pre‐poled in the thickness direction. Although the PVDF modulus is 3.6 GPa, our previous studies revealed that orders of magnitude reduction in stiffness and maximum intrinsic strain could be achieved when stiff films are patterned into serpentine ribbons.^23^ Of course, there is a tradeoff between softness/stretchability and sensitivity as modeled in our recent paper,^24^ we will be studying this tradeoff carefully. We have also developed a cost‐effective cut‐and‐paste method to pattern thin films such as metalized polymer sheets^10^ as well as polymer‐supported 2D materials^25^ into serpentine shapes. By applying the cut‐and‐paste method to produce FS PVDF mesh, the ultrathin piezoelectric e‐tattoo we created has a stretchability of more than 110% with a sensitivity of 0.4 mV per microstrain (*µε*). To guide and validate the FS PVDF SCG sensor, a 3D digital image correlation (DIC) method was used to map the motion of the chest derived from respiration and the cardiac movement. If respiration induced movement can be decoupled from the cardiac movement induced chest vibration, the map can help identify the optimal sensing spot to mount the SCG e‐tattoo. Furthermore, strain mapping of the chest with different types of SCG sensors can determine how the mechanical stiffness of the sensor substrate affects the sensitivity of the e‐tattoo. As piezoelectric sensors are also susceptible to motions other than the cardiac movement, we also developed a dual PVDF sensor system for noise subtraction to combat the motion artifacts.

Our cut‐and‐paste fabrication method allows easy and rapid patterning and integration of multiple materials in one tattoo. By integrating stretchable PVDF vibration sensor with stretchable gold electrodes, we will present a dual‐mode soft e‐tattoo for synchronous electro‐ and mechano‐acoustic cardiovascular (EMAC) sensing. The EMAC tattoo does not contain any rigid components and has a dimension of 63.5 mm × 38.1 mm × 0.122 mm, a total mass of 150 mg, a mass density of 0.5 g m^−3^, an effective modulus of 8.5 MPa, and a stretchability of 100%, which constitutes the thinnest and lightest EMAC sensing platform ever reported. It can be applied conformably and unobstructively on the human chest without acoustic impedance mismatch with the skin. The EMAC e‐tattoo can measure high fidelity ECG and SCG simultaneously and synchronously, from which a rich variety of characteristic cardiac time intervals can be extracted. We found that among those time intervals, the systolic time interval (STI) has a strong negative correlation with blood pressure (BP). This prompted us to estimate BP beat‐to‐beat using the EMAC e‐tattoo.

## 2 Results

### 2.1 SCG Measured by Stretchable Piezoelectric Tattoo, Accelerometer, and 3D DIC Method

**Figure 1:**
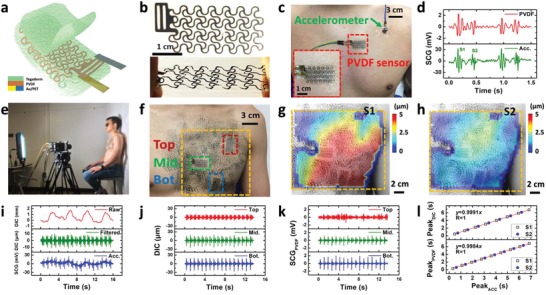
Stretchable PVDF vibration sensor (i.e. PVDF e‐tattoo) and 3D DIC method for SCG measurement. a) A schematic of the stretchable PVDF e‐tattoo. b) Photographs of an undeformed and stretched PVDF e‐tattoo. c) The PVDF e‐tattoo (red boxed) and a commercial accelerometer (green arrowed) attached on human chest. d) SCG signals measured by the PVDF e‐tattoo and the accelerometer. e) A photograph of the 3D DIC setup for mapping human chest deformation. f) A photograph of a human chest mounted with three PVDF e‐tattoos and painted with a random speckle pattern. Positions of the three e‐tattoos are denoted as Top, Mid., and Bot. g,h) The out‐of‐plane displacement map averaged at S1 and S2 peak times, respectively. i) Measured signals by 3D DIC method (raw, filtered) and the accelerometer (Acc.) from the chest. j,k) SCG signals at three different positions (Top, Mid., and Bot.) captured by 3D DIC method and PVDF e‐tattoos, respectively. l) The correlation of SCG peak times (S1 and S2) measured by the 3D DIC method, PVDF e‐tattoo, and the accelerometer.

The SCG e‐tattoo is an ultrathin and stretchable vibration sensor based on a FS network of PVDF. A schematic of the SCG e‐tattoo is shown in **Figure** **1**a. A 28‐µm‐thick PVDF film with 80‐nm‐thick Cu‐Ni electrodes on the top and bottom surfaces (piezo film sheets, TE Connectivity) was patterned into a FS mesh by a mechanical cutter plotter (Cameo, Silhouette) within minutes. The total size of the PVDF mesh is 38.1 mm × 18.1 mm, and the in‐plane waviness defined by the serpentine width‐to‐radius ratio (*w*/*R*) is 0.4, with *w* = 0.5 mm and the joint angle α = 15°. The use of *w*, *R*, and α is following a convention of geometric parameters for serpentines.^23^ In contrast to our previous cut‐and‐paste method which used thermal release tapes,^10^ a weakly adhesive transfer tape (TransferRite Ultra 582U, American Biltrite Inc.) was used as the temporary support to avoid thermal stress in PVDF. After patterning, the stretchable PVDF mesh was sandwiched by two 47‐µm‐thick stretchable medical tapes (Tegaderm, 3M) to avoid discharge through human skin. The whole cut‐and‐paste process (Figure S1, Supporting Information)^10^ is chemical‐ and mask/stencil‐free and can be completed within 20 min. Figure 1b presents a photograph of the PVDF e‐tattoo without and with tension. The FS network ensures both stretchability and compliance. To validate its functionality as an SCG sensor, we compared the PVDF e‐tattoo with an accelerometer with a sensitivity of 100 mV g^−1^ (Model 352C65, PCB Piezotronics). Both the e‐tattoo and the accelerometer were attached to the chest of a human subject as displayed in Figure 1c. Filtered SCG signals simultaneously captured by the PVDF e‐tattoo and the accelerometer (dorsal–ventral direction) are plotted in Figure 1d, which shows comparable waveforms and well‐aligned peaks labeled as S1 and S2, where “S” stands for sound.

The location dependency of SCG has been debated discussed by several studies, yet no consensus has been reached. Most researchers placed accelerometers on the sternum,^26^ while other studies suggested that left midsternal region could provide much stronger signals.^27^ Lin et al. detected distinctive SCG waveforms in four different cardiac valve auscultation sites.^28^ Although SCG monitoring using multiple sensors was also proposed,^29^ the spatial resolution from a sensor array is not sufficient to generate a high‐quality mapping, and the installation of a sensor array is arduous. Herein, we propose to use 3D DIC, a noncontact, high resolution, full‐field surface deformation measurement method, to map chest vibration due to SCG and to identify the best location for SCG sensors. In fact, 3D DIC has been frequently used to investigate the mechanical properties of biological tissues^30^ and skin deformation of human body.^31^ But this is the first time that 3D DIC is applied for SCG measurement.

To carry out the 3D DIC measurement, we painted the chest of an adult male with a random speckle pattern and set up two high‐speed cameras (Phantom Miro 310, AMETEK) to focus on the chest at an angle of 30° (Figure 1e). The cameras captured the surface of the chest for 16 s at 500 Hz frame rate while the subject was sitting still and breathing normally. Figure 1f shows the randomly speckled chest surface with a yellow box indicating the region of interest captured by the high‐speed cameras. Three independent PVDF e‐tattoos were attached at three different locations on the chest to determine the location dependency of SCG. They were oriented in the “radial” directions based on the postulation that the chest surface “inflates” and “deflates” similar to a balloon. The averaged out‐of‐plane displacement maps at S1 and S2 peak times are displayed in Figure 1g,h. The corresponding 3D displacement maps of one cardiac cycle are shown in Figure S3 (Supporting Information). Although different subjects showed slightly different peak locations for S1 and S2 (Figure S4a and S4b, Supporting Information), in general, we found that S1 is significant (≈5 µm) around the left nipple area, while S2 appears to be strong (≈3 µm) at a location much higher than the left nipple. Such location‐specific amplitude is consistent with the cardiac valve auscultation sites as S1 is induced by mitral valve closure (MC) and S2 comes from aortic valve closure (AC).^32^, ^33^

The raw and filtered out‐of‐plane displacement at a point near the top e‐tattoo measured by DIC and the chest vibration near this point measured by the accelerometer are plotted together in Figure 1i for comparison. The raw displacement (red curve on the top of Figure 1i) contains both breathing and heartbeat induced chest vibrations (Figure S5, Supporting Information), but is dominated by breathing with an amplitude of ≈1 mm at a frequency ≈0.3 Hz. To extract the SCG signal from the raw displacement, a 4th order Butterworth filter with 12–40 Hz bandwidth was applied, which yielded the green curve in the middle of Figure 1i. Compared with the SCG measured by the accelerometer (blue curve at the bottom of Figure 1i), the DIC‐measured SCG shows almost identical S1 and S2 peaks. Thereby, we have proved that the SCG signal could be extracted from the 3D DIC measurement even though it is only a few micrometers embedded in the millimeter‐range breathing signals.

The location‐dependent SCGs are revealed by both the DIC measurement (Figure 1j) and the e‐tattoo measurement (Figure 1k). Figure 1j plots the out‐of‐plane displacements at three different chest locations close to the three e‐tattoos highlighted in Figure 1f. Figure 1k plots the piezoelectric output from the three e‐tattoos. It is clear that Figure 1j,k are comparable regarding the relative amplitude of S1 and S2 at different locations—S1 is the strongest at the bottom e‐tattoo and reasonably strong at the middle e‐tattoo while S2 is visible at the top and middle e‐tattoos but not the bottom one. Such an observation is consistent with the S1 and S2 maps offered in Figure 1g,h, respectively. In this regard, the middle e‐tattoo is the best location for SCG as both S1 and S2 are distinctively measurable.

The correlation of S1 and S2 peak times measured by DIC, e‐tattoo, and accelerometer is revealed in Figure 1l. The top panel plots the peak times measured by DIC as functions of those measured by accelerometer and the bottom panel is e‐tattoo versus accelerometer. Perfect correlation was found in both plots, confirming that all three methods are valid for SCG measurements. But considering the DIC method is not mobile and accelerometer is rigid, PVDF e‐tattoo exhibits some unique advantages such as thinness, softness, wearable, and passive sensing. In the following sections, we will investigate the PVDF e‐tattoo performance in detail.

### 2.2 PVDF E‐Tattoo Characterization

**Figure 2:**
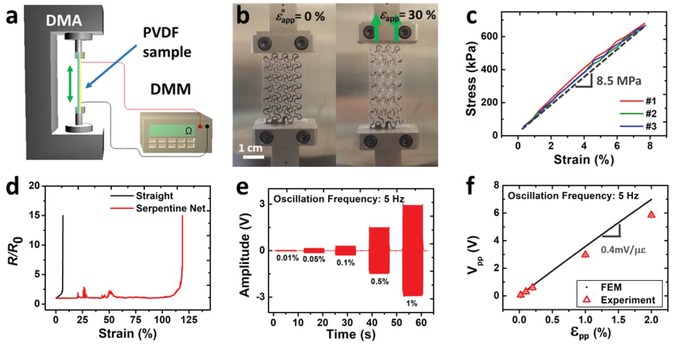
Electromechanical characterization of the PVDF e‐tattoo. a) A schematic of a PVDF e‐tattoo subjected to tensile test with in situ electrical measurements. b) Photographs of a PVDF e‐tattoo under tensile test, from 0% to 30%. c) Stress–strain curves of three different PVDF e‐tattoos, whose slope suggests the effective modulus to be 8.5 MPa. d) The measured electrical resistance versus tensile strain curves indicate the stretchability of a straight PVDF ribbon to be 5.8% (black) and a filamentary serpentine (FS) PVDF network to be 112.9% (red). e) Voltage output from the FS PVDF e‐tattoo under 5 Hz sinusoidal strain amplitude from 0.01% to 1%. f) Experimental (red) and FEM (black) results of compensated peak‐to‐peak voltage output with respect to peak‐to‐peak applied strains.

According to the manufacturer datasheet (Table S1, Supporting Information), the in‐plane Young's modulus and strain sensitivity of the as‐purchased PVDF film are 2–4 GPa and 12 mV per microstrain (*µε*), respectively.^17^ To obtain accurate material properties, we performed our own mechanical and electromechanical characterizations using a RSA‐G2 dynamic mechanical analyzer (DMA) and a Rigol digital multimeter (DMM), as illustrated in **Figure** **2**a. To measure the electromechanical response of PVDF, we adopted the oscillation mode of the DMA and conducted a cyclic test. Meanwhile, the electrical output of the PVDF sample was collected by an NI DAQ (NI‐6225, National Instruments). Since the PVDF sensor has a high output impedance, a voltage follower (Figure S6b, Supporting Information) was connected between the PVDF sensor and the DAQ. A 1 GΩ resistor was placed right before the voltage follower to remove the low‐frequency fluctuation by coupling with the capacitance of PVDF. Details of the characterization methods can be found in the Experimental Section. It is important to mention that the PVDF is uniaxially stretched during the manufacturing process. This makes it electrically orthotropic in‐plane; however, it remains mechanically isotropic under small strains.^34^ We confirmed that the PVDF film in‐plane Young's modulus is 3.6 GPa in both directions (Figure S6a, Supporting Information). Its electromechanical sensitivity was measured to be 11.1 mV per *µε* in the stretching direction but only 1.1 mV per *µε* in the transverse direction, which was not available in the datasheet.^17^ Therefore, when the FS PVDF is patterned with longitudinal direction aligned with the stretching direction as shown in Figure S6c, it is more sensitive to strains in the longitudinal direction than the transverse direction.

Properties above were measured for unpatterned PVDF sheets. After patterning the sheet into a FS network, the mechanical and electromechanical behavior will change drastically. In the following paragraphs, we will characterize and model the PVDF e‐tattoo, which consists of a FS PVDF network on a 3M Tegaderm tape. The modulus, stretchability, and electromechanical sensitivity were measured using the setup displayed in Figure 2a,b. Figure 2c presents the strain–stress curve of the FS PVDF e‐tattoo and the effective modulus *E*_1_ is determined to be 8.5 MPa, which is only 0.24% of the unpatterned PVDF membrane. Stretchability can be determined by the critical strain at which the electrical resistance increases drastically during stretch.^35^ Accordingly, Figure 2d shows that the FS PVDF has a stretchability of 112.9%, which is ≈20 times higher than that of a straight PVDF ribbon (5.8%). In addition, a device with such structure can be stretched about 10 000 times at 20% applied strain without losing its functionality.^10^

Although FS PVDF demonstrates an enhanced compliance and stretchability compared with its linear counterparts, its electromechanical sensitivity is compromised. We measured the output voltage across the thickness direction at different strain levels. Figure 2e plots the amplitude of the output voltage generated from an FS PVDF when stretched by five different strain amplitudes, ranged from 0.01% to 1%, at 5 Hz oscillation. Plotting measured peak‐to‐peak voltage (*V*_pp_) as a function of peak‐to‐peak applied strain (ε_pp_) in Figure 2f as red open markers indicates a linear relationship between the two.

To fully understand the electromechanical behavior of piezoelectric serpentines, we also performed finite element modeling (FEM) using the commercial FEM software ABAQUS. The modeling procedure is described in the Experimental Section, and the input material properties were those measured in‐house. The output voltage can be calculated based on the stress distribution using the following equation

(1) $$V=\frac{h}{\varepsilon_{3}A}\int_{A}d_{3j}\sigma_{j}dS$$

where *A* represents the total area covered by the electrodes, *h* is the thickness of the PVDF film, and ε_3_ is the out‐of‐plane permittivity of the PVDF film, which was calculated to be 90.31 pF m^−1^ based on our capacitance measurement. Plotting the measured raw and properly compensated peak‐to‐peak output voltages (red) along with those independently computed by FEM (black) in Figure 2f, we found a good agreement between the two. The slope of Figure 2f indicates the electromechanical sensitivity of the FS PVDF e‐tattoo to be 0.4 mV per *µε*, which is only 3.6% of the sensitivity in the stretching direction and 36% of that in the transverse direction of straight PVDF ribbons. As a piezoelectric material, PVDF generates electrical charges out of mechanical stress. Since a serpentine is a well‐known stress‐relieving structure, the voltage output is expected to be much lower than its linear counterpart. Therefore, high compliance and stretchability of the FS PVDF mesh are achieved at the cost of electromechanical sensitivity.

For the application of measuring SCG by a tattoo‐like vibration sensor attached to the chest, high compliance of the sensor is essential to reduce mechanical constraint, chaffing, and acoustic impedance mismatch on the skin. Therefore, we adopted the FS PVDF as our SCG sensor for its softness, stretchability, and low mass density, which are comparable to the stratum corneum of human skin.^36^ Despite the compromised sensitivity, the FS PVDF e‐tattoo can measure high‐fidelity SCG from the chest, as confirmed in Figure 1k,l.

### 2.3 Substrate Effects on Skin Deformation and Device Sensitivity

**Figure 3:**
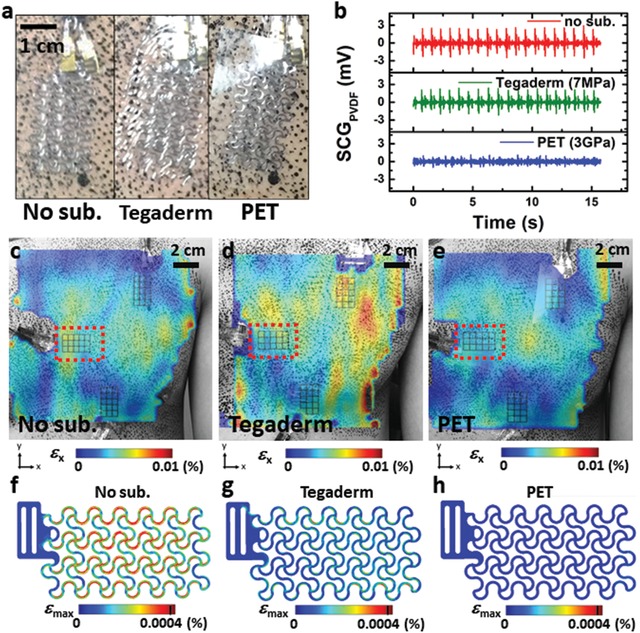
Substrate effects on FS PVDF vibration sensors. a) Photographs of sensors with no substrate, 47‐µm‐thick Tegaderm, and 50‐µm‐thick PET covered over the PVDF. b) SCG signals recorded by the three different types of PVDF sensors at the Mid. location. The 12–40 Hz range, time‐averaged maximum principal strain maps of c) no substrate case, d) Tegaderm case, and e) PET case. FEM results of maximum principal strain in the FS PVDF with f) no substrate, g) Tegaderm substrate, and h) PET substrate.

If the FS PVDF sensor is supported by a stiff substrate, it is expected to constrain skin deformation and lower the sensitivity of the sensor. To investigate the effects of the substrate stiffness, the 3D DIC method was used again to visualize maximum principal strains on skin surface. **Figure** **3**a shows three representative types of FS PVDF sensors: no substrate, 47‐µm‐ thick Tegaderm (modulus of 7 MPa), and 50‐µm‐thick polyethylene terephthalate (PET) (modulus of 2.5 GPa). A thin layer (≈1 µm) of rosin‐based adhesive (De‐Hesive Spray, Cramer) was applied between all the sensors and the skin for mechanical adhesion and electrical insulation. Three DIC measurements were carried out for the three different types of sensors. For each DIC measurement, three FS PVDF sensors of the same type were attached to three specific locations: top, middle, and bottom as labeled in Figure 1f. Synchronous DIC and PVDF measurements were carried out for 16 s at 500 Hz. Figure 3b plots the SCG signals captured by the three different types of the PVDF sensors at the Middle location. It is evident that the sensor without a substrate exhibits the largest SCG amplitude, followed by the sensor with a Tegaderm substrate. The PVDF sensor with PET substrate could not measure a useful SCG signal because the in‐plane strains of the chest are barely transferred to the sensor when the sensor is significantly stiffer than human skin.^37^

Strain maps obtained by 3D DIC can directly quantify the substrate effects. Figure 3c–e displays the maximum principal strain maps within the frequency range of SCG (12–40 Hz). It is evident that the strain over the no‐substrate sensor blends in with the natural strain fields of the skin whereas the PET‐supported sensor shows clear mechanical constraints on the skin.

The substrate effects on device sensitivity were further investigated by FEM. For each type of substrate, the measured SCG induced peak displacements of the Middle sensor was entered in an FEM model as the boundary conditions. The maximum principal strain distribution of the three different types of FS PVDF sensors is depicted in Figure 3f–h, from left to right: no substrate, Tegaderm, and PET. Without any fitting parameter, our FEM estimated the corresponding output voltages of the FS PVDF sensors to be 2.74, 2.20, and 0.86 mV, respectively, which agree well with the experimentally measured voltage outputs shown in Figure 3b. This finding proved that first, SCG induced chest surface strain is only a few micro strains; second, we fully understood the substrate effects and hence are able to predict the sensor output given substrate stiffness. In summary, the substrate for the FS PVDF vibration sensor should be as soft as possible to allow for skin deformation because its sensitivity would diminish otherwise. Although the FS PVDF sensor with no substrate demonstrates the best sensitivity, it may not be the easiest to handle and apply. Therefore, we choose the Tegaderm‐supported FS PVDF sensor as the vibration sensing e‐tattoo considering its sensitivity is only 20% lower but much easier to use.

### 2.4 Dual‐PVDF‐Sensor for Motion Artifact Cancellation

**Figure 4:**
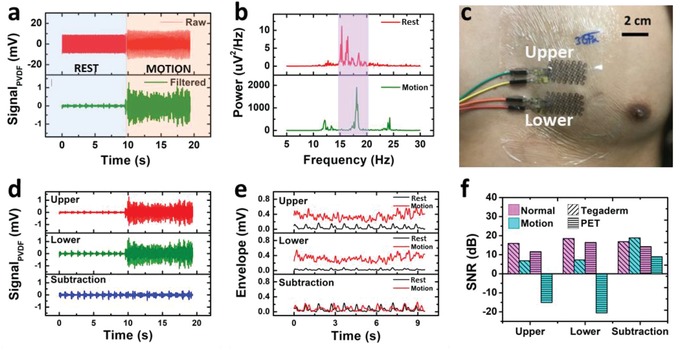
Dual‐sensor‐based motion artifact cancellation scheme. a) Raw and filtered SCG signals captured by one PET‐covered PVDF sensor under rest (blue shaded) and motion (yellow‐shaded) conditions. b) Periodograms of the filtered SCG signals under rest and motion conditions. c) A photograph of the dual sensor system attached on human chest. d) Filtered SCG signals and e) corresponding envelopes recorded by upper and lower PVDF sensors and their subtraction result under normal and motion conditions. f) Signal‐to‐noise ratios (SNR) of single and dual sensor systems with different substrates (PET vs Tegaderm) under normal and motion conditions.

Although we have demonstrated that a single PVDF e‐tattoo can pick up both S1 and S2 from the “middle” position while the subject stays still, the piezoelectric sensor is, however, sensitive to all kinds of body motion. In principle, motion artifacts or power noises outside of the signal frequency range are easy to remove by filtering. The section labeled as “REST” in **Figure** **4**a shows an example of signal processing. Corrupted by the 60 Hz power noise, the SCG waveform is unrecognizable from the raw signal measured by the FS PVDF sensor. In this case, the SCG waveform can be extracted from the raw signal using a band‐pass filter from 12 to 40 Hz. However, noise within such frequency range is almost inseparable by conventional filters. As observed in the section labeled as “MOTION” in Figure 4a, during which time the subject was shaking his left leg. In this case, the motion artifacts can no longer be removed with the same filter. The phenomenon can be explained by Figure 4b. After applying fast Fourier transform (FFT) to the filtered “REST” and “MOTION” data, the peak power of the “MOTION” data falls in the frequency band of SCG, with 200 times higher power than that of the SCG signal.

Different approaches have been introduced to resolve this issue through digital processes such as smoothing,^38^ adaptive filtering,^39^ threshold algorithm,^40^ etc. Recently, Yang et al. suggested a hardware approach to cancel the motion artifact using a dual‐accelerometer system.^39^ According to this paper, when two accelerometers are placed at different locations on the chest, SCG detected from the two accelerometers are slightly different, while motion artifacts from two accelerometers are highly identical. When signals measured by the two accelerometers are subtracted, the motion artifacts could be effectively reduced such that the SCG features can emerge. From our DIC results, we've also found that the SCG waveforms vary with locations on the chest, which is a visual confirmation of such findings. Instead of using accelerometers, we attached two FS PVDF sensors (“upper” and “lower”) 1 cm apart near the Middle position for motion cancellation as shown in Figure 4c. A muscle‐stimulating device (Rechargeable TENS Unit Muscle Stimulator, AUVON) was attached to the left leg of the subject to generate a 10 s consistent leg shaking motion of which the frequency is within 12–40 Hz, which overlaps with the frequency range of SCG.

Figure 4d plots signals measured by the two PVDF sensors and their difference during the experiment, all of which are after the band‐pass filter (12–40 Hz). Compared to signals from individual sensors, the differential signal shows the cleanest SCG waveforms during the motion period. Signal envelopes are plotted in Figure 4e: the upper sensor in the top panel, the lower sensor in the middle panel, and the subtracted signal in the bottom panel. In each panel, the SCG envelop during the rest period is plotted in black and during motion is plotted in orange. It is obvious that the rest and motion envelopes are drastically different for individual sensors (top and middle panels) but become very comparable in both amplitude and frequency after subtraction (bottom panel). The performance of the single and the dual sensor systems can also be quantitatively evaluated by signal‐to‐noise ratio (SNR) offered in Figure 4f. We use *S*_r_ and *S*_m_ to denote the power of the actual SCG signal within 12–40 Hz during the REST and MOTION period, respectively. Then we know *S* = *S*_r_ = *S*_m_ as the two SCG signals were recorded from a single experiment. Here we assume the REST period does not have any noise within 12–40 Hz while the MOTION period has the noise within 12–40 Hz. Thus, the pure *S* can be derived from calculating the power of the rest period within 12–40 Hz. Then, the noise power *N*_i_ and the SNR value of each period can be calculated as follows

(2) $$\{\begin{array}{c} N_{i}=P_{i}-S\;\;\;\;\;\\ {\mathrm{SNR}}_{i}(\mathrm{dB})=10\log_{10}\left(\frac{S}{N_{i}}\right) \end{array}\;i=r,m$$

where *P*_i_ is the total power of each period. The SNRs during REST and MOTION periods for individual sensors with Tegaderm and PET substrates and the dual sensor system are plotted together in Figure 4f for comparison. At the individual sensor level, it can be easily concluded that: i) at a given period (REST or MOTION), sensors with Tegaderm substrate have a higher SNR than sensors with PET substrate (see Figure S7, Supporting Information for more details), which can be attributed to the substrate effects as discussed in Figure 3; ii) signals measured during MOTION periods always suffer from significantly lower SNRs compared with REST periods; iii) the negative SNR for a single sensor with PET substrate under motion indicates that SCG is undetectable in this case. Here the result of the dual sensor noise cancellation with PET substrate was provided instead of with Tegaderm substrate to prove the dual sensor system can restore the SCG signal even for the severe motion noise due to the stiff substrate. The performance of the dual sensor system is distinctly different in the sense that the SNRs during the MOTION periods can be comparable with that during the REST periods. In conclusion, because of the location‐dependent nature of SCG, differential SCG signal using a dual sensor system exhibits reduced motion artifacts compared to an individual sensor. This study hence paved the way for ambulatory SCG monitoring using soft e‐tattoos.

### 2.5 EMAC Sensing E‐Tattoo

**Figure 5:**
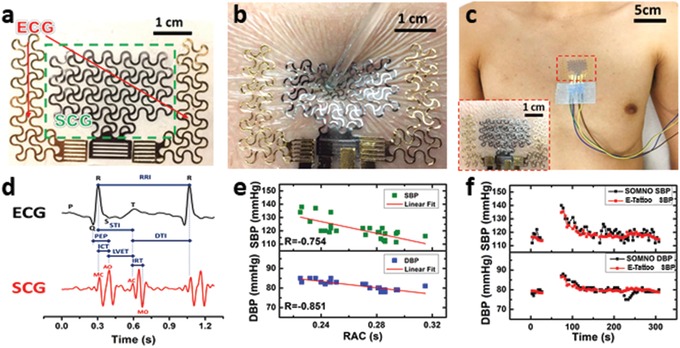
Stretchable EMAC sensing tattoo for continuous BP estimation. a) A photograph of the EMAC tattoo that houses an FS PVDF vibration sensor and a pair of FS Au electrodes. b) Tattoo‐like behavior of ultrathin, stretchable EMAC on human skin. c) A photograph of an EMAC sensing tattoo on human chest with wire connections. d) Synchronously measured ECG (black) and SCG (red) by EMAC tattoo after filtering with a variety of cardiac time intervals illustrated. e) The negative correlation between SBP/DBP measured by SOMNOtouch and RAC measured by an EMAC sensing tattoo for one subject. f) Continuous SBP and DBP estimated by SOMNOtough (black) and EMAC tattoo (red) during Valsalva maneuver.

Integrating a pair of FS Au electrodes and one FS PVDF vibration sensor on one piece of Tegaderm can result in an EMAC sensing e‐tattoo of much higher medical value than the individual sensors. We designed the FS Au electrodes (100 nm thick Au on 13‐µm‐thick PET) to have the same serpentine characteristics as the FS PVDF vibration sensor. Two Au electrodes 3 cm apart can be first cut‐and‐pasted on the Tegaderm followed by cutting‐and‐pasting the FS PVDF in between the Au electrodes. The detailed manufacturing process is illustrated in Figure S8 (Supporting Information). A picture of the EMAC e‐tattoo is presented in **Figure** **5**a. The in‐plane dimension of the tattoo is 63.5 mm × 38.1 mm, the total thickness, including the double layer Tegaderm, is 122 µm, and the total mass is 150 mg excluding FFC. The stretchability of the EMAC e‐tattoo, limited by the FS Au/PET, is about 100% (Figure S9, Supporting Information), while the effective modulus is 8.5 MPa, similar to the FS PVDF vibration sensor. When laminated on human skin, its deformation was compatible with the skin just like a temporary tattoo as illustrated in Figure 5b. Even after severe skin deformation, the device remained fully functional without delamination, slippage, or mechanical failure.

The stretchable EMAC e‐tattoo can be mounted on human chest as shown in Figure 5c. The inset shows a magnified view of the tattoo on the skin. We placed the tattoo according to our previous discovery in Figure 3 that the left edge of the middle of the sternum offers both reasonable S1 and S2.^26^ Figure 5d displays synchronously measured ECG (black) and SCG (red) signals by the tattoo after signal processing. The DAQ and signal processing will be described with more details in the next section. The P, Q, R, S, and T peaks of the ECG, and the MC, AO (aortic valve opening), AC (aortic valve closing), and MO (mitral valve opening) peaks of the SCG waveforms are labeled in Figure 5d. A Wiggers diagram (Figure S10, Supporting Information), which illustrates major cardio‐physiological events and their corresponding peaks in ECG and PCG,^41^ clearly tells that the R peak of the ECG is the signature of the closure of the mitral valve^32^ and the onset of 2nd PCG feature reflects the closure of the aortic valve, which is identical to the AC peak of SCG.^33^ In Figure 5d, we find that the R peak of the ECG and the MC peak of the SCG are almost aligned, but the R peak is much easier to extract as it is the highest peak in ECG whereas the MC peak is neither aligned with S1 nor S2. Many characteristic times and intervals of the cardiac mechanics can be determined from this plot.^32^, ^33^ From the SCG alone, we can determine the isovolumetric contraction time (ICT) to be the MC‐AO interval, the left ventricle ejection time (LVET) to be the AO‐AC interval, and the isovolumic relaxation time (IRT) to be the AC‐MO interval. Looking at the two cardiograms synergistically, more features can be extracted. For example, the Q‐AO interval represents the pre‐ejection period (PEP), i.e., the period between when the ventricular contraction occurs and the semilunar valves open and blood ejection into the aorta commences. Moreover, the systolic time interval (STI) and the diastolic time interval (DTI) can be extracted as the R‐AC and AC‐R intervals, respectively. Some of these time intervals are related. For example, it is clear from the chart that STI = ICT + LVET. Among the aforementioned time intervals, we will focus on the R‐AC interval, i.e., the STI, for its reported correlation with BP.^42^

### 2.6 Correlation between Hemodynamic Parameters and BP

For decades, biomedical engineers are in search of methods to monitor BP continuously but noninvasively.^43^ In addition to the well‐known pulse transit time (PTT) method,^43^ new methods for continuous BP estimation using bioimpedance sensors,^44^ and wearable ultrasound sensors^45^ are also emerging in recent years. However, all previous sensors involved rigid components such as MEMS accelerometers, rigid metal electrodes, and piezoelectric ceramics. Herein, we want to demonstrate that our soft EMAC sensing tattoo is capable of continuous BP estimation. So far, quite a few papers have mentioned the correlation between the STI and BP.^13^, ^46^, ^47^, ^48^, ^49^ Tang and co‐workers have noticed a strong negative correlation between systolic blood pressure (SBP) and systole through measurements on six subjects using a conventional ECG sensor and a microphone for PCG. They stated that exercise elevated the heart rate and cardiac muscle contractility in the first phase, which would result in shorter valve closure time intervals.^48^ In later work, they conducted animal experiments to investigate the correlation between the systole (RAC) and the blood pressure of the left ventricle (LVBP).^49^ The variation of BP, which ranged from 90 to 282 mmHg, controlled by different doses of epinephrine, was measured by an invasive catheter inserted into the left ventricle. The result indicated that RAC and SBP are highly correlated, and the correlation can be used for accurate, continuous, and noninvasive SBP estimation. In addition, strong correlations between the individual BP and the duration of systole in 16 subjects were found using a custom stethoscope for both ECG and PCG, according to Zhang's paper.^47^ They attributed the correlation to the change in peripheral resistance, heart rate, and contractility of the heart.^47^ Nevertheless, these mechanoacoustic sensors were neither wearable nor stretchable.

Here, we demonstrate that the EMAC e‐tattoo is able to capture the RAC interval for continuous BP estimation. For calibration and validation, we adopted a commercially available PTT‐based noninvasive continuous BP measurement device, the SOMNOtouch NIBP. To vary the subject's BP abruptly, we adopted a procedure named the Valsalva maneuver, which is a half‐minute operation during which the test subject makes a forceful attempt of exhalation with closed mouth and nose. Dynamic variation of BP and HR can be observed during such tests.^50^ In our experiment, each subject was asked to perform the Valsalva maneuver in a sitting posture. The SOMNO and the e‐tattoo data acquisition was started simultaneously. After 30 s of rest with normal breathing, the subject executed the Valsalva maneuver for 30 s, followed by a minute of relaxation with normal breathing. Several Valsalva maneuver experiments were repeated with at least a 10‐min break in between for each subject. During those experiments, beat‐to‐beat SBP and diastolic blood pressure (DBP) were measured by SOMNO while beat‐to‐beat RAC intervals were measured by our EMAC e‐tattoo via LabView.

Four subjects were recruited for the tests, and the result from one of the four is displayed in Figure 5e,f. Figure 5e shows the linear correlation between BP and RAC. Pearson's correlation coefficients are labeled as R in the plots. Our results suggest that both SBP and DBP have reasonable negative correlations with RAC, but the fitting parameters and correlation coefficients vary from individual to individual and also from posture to posture. After obtaining the calibration equation for each subject, we can calculate their BP out of the measured RAC in independent Valsalva maneuver experiments. Figure 5f presents BP estimated by the EMAC e‐tattoo (red) versus SOMNO (black). Data during the Valsalva maneuver was omitted as both the SOMNO and SCG signals suffered from excessive noise induced by body shivering, as evident in Figure S11 (Supporting Information). When the subject is at rest, Figure 5f shows reasonably good agreement between the SOMNO and the tattoo. Recognizable mismatches are likely derived from motion or slight posture change. Results from other three subjects are offered in **Table** **1** and Figure S12 (Supporting Information).

**Table 1:** Pearson's correlation coefficients for three different pairs of parameters of four different human subjects

Subject	SBP, RAC	DBP, RAC	PBP, RAC
Subject 1	−0.754	−0.851	−0.618
Subject 2	−0.672	−0.845	−0.24
Subject 3	−0.814	−0.863	−0.471
Subject 4	−0.848	−0.872	−0.823
Average	−0.772	−0.858	−0.538

To determine if there is a unified correlation between BP and RAC for a group of people, we compiled data for all four subjects in Figure S13 (Supporting Information) and listed their *R* values in Table 1. Both the compiled SBP to RAC and the compiled DBP to RAC show a correlation. We also confirmed that the heart rate (HR; Figure S14, Supporting Information) and the pulse blood pressure (PBP), which is the difference between SBP and DBP, is less pertinent to RAC. Note that we could not derive master fitting equations for BP estimation from the compiled SBP‐RAC plot as there is a huge difference between the overall and individual fitting parameters. Thus, the inference is that there is a unique relationship between RAC and BP for each individual at a given posture, and calibration for each individual will be necessary, just like the SOMNOtouch NIBP.

## 3 Discussion

A hair thin, skin soft, and highly stretchable PVDF vibration sensing e‐tattoo is created for high fidelity SCG monitoring. Using the 3D DIC method, the chest deformation was fully mapped and analyzed. Based on the displacement map at S1 and S2 peaks, we found the left edge of middle of the sternum is the optimal spot for SCG sensors. The study on the substrate stiffness reveals that a low elastic mismatch between human skin and the e‐tattoo is desirable for high sensitivity. We also proved that motion artifacts are removable through a dual sensor setup by virtue of the inhomogeneity of SCG waveforms. Integrating Au electrodes and PVDF vibration sensors on one substrate, we created an e‐tattoo for electro‐mechanoacoustic cardiovascular (EMAC) sensing, which can synchronously measure ECG and SCG. Such synchronous measurement affords the extraction of many characteristic cardiac time intervals among which the systolic time interval (STI) is found to have a strong negative correlation with BP. Hence the EMAC sensing tattoo also offers a possibility for continuous and unobstructive BP estimation. This stretchable and wearable EMAC sensing tattoo may also be useful in other medical settings where mechanoacoustic signatures are important, such as obstructive sleep apnea. Moreover, the EMAC e‐tattoo can be potentially used for measuring systole during resting and exercise to diagnose coronary artery disease.^51^

In future work, wireless devices such as NFC or Bluetooth would be integrated which allow the EMAC sensing tattoo to be used for practical ambulatory applications.

## 4 Experimental Section

*Fabrication of Filamentary Serpentine PVDF Vibration Sensor*: Figure S1 (Supporting Information) illustrates the fabrication procedure using the cut‐and‐paste method,^10^ which constitutes four major steps: i) placing the as purchased PVDF film on a weak adhesive transfer tape; ii) cutting the film by a mechanical cutter plotter; iii) removing extraneous parts; iv) transferring remaining filamentary serpentine (FS) mesh to a target adhesive substrate. A more detailed procedure is described as follows. An electroded 28.4 µm thick PVDF film (piezo film sheets, TE connectivity) was attached to a 100 µm thick weak adhesive transfer tape (TransferRite Ultra 582U, American Biltrite Inc.) backed by a supporting film (350 GSM, Inkpress Media) (Step 1). Within several minutes, the PVDF film was carved by a mechanical cutter plotter (Cameo, Silhouette) with designed patterns. For optimal cutting quality, the blade setting in the software (Silhouette Studio) was established with 5 in blade exposure, 1 in cutting speed, and 3 in depth. Due to the weak adhesion of the transfer tape, extraneous parts of the PVDF film can be manually peeled off by tweezers after the cutting, leaving just the FS mesh on the weak adhesive transfer tape (Step 2). Then, the mesh was transferable to the target substrate (Tegaderm tape, 3M), due to the significantly different adhesive forces of the tapes: 2.2 N/25 mm @ 90° for the transfer tape and 35.6 N/60 mm for the Tegaderm tape (Step 3). Before and after the FS PVDF was transferred, a pair of rectangular Au/PET connectors in the size of 25.4 mm × 3.81 mm backed by a self‐adhesive laminating sheet (Avery) was attached to the Tegaderm to extend the top and bottom electrodes of the PVDF for the connection with a flat flexible connector (FFC, Clincher Flex Connectors, Amphenol FCI) (Step 4). Finally, a 2nd Tegaderm layer was applied to encapsulate just the FS PVDF to prevent the direct contact of the PVDF electrode with human skin (Step 5).

*Fabrication of EMAC Sensing Tattoo*: Figure S8 (Supporting Information) illustrates the fabrication procedure for the EMAC sensing tattoo. A 76.2 mm × 50.8 mm large 100 nm Au on 12.5 µm PET bilayer was cut‐and‐pasted on the Tegaderm tape (Steps 1–4). Then, an Au/PET connector was placed to the Tegaderm with Au facing up (Step 5) for the bottom electrode extension of the to‐be‐pasted FS PVDF. In the same manner above, a FS PVDF was patterned from an electroded PVDF film (Steps 6 and 7). After the FS PVDF sensor was transferred (Steps 8 and 9), three more Au/PET connectors were added such that the two ECG electrodes and the top electrode of PVDF can be connected to the FFC (Step 10). Finally, the FS PVDF was encapsulated by a 2nd Tegaderm layer (Step 11). Dimension of the final EMAC e‐tattoo was 63.5 mm × 38.1 mm × 0.122 mm.

*Measuring PVDF Elastic Properties*: The PVDF datasheet provides the in‐plane elastic stiffness constant (*C*_1_) of PVDF to be 2–4 GPa. To obtain an accurate value of *C*_1_ and to examine whether there is orthotropy in stiffness, uniaxial tensile tests were carried out using a RSA‐G2 DMA. Two sets of straight and freestanding PVDF ribbons of size 36 mm × 2.25 mm were prepared by cutting along the two orthogonal directions of the commercial PVDF sheet. Each set, containing three ribbons, was subjected to five cycles of uniaxial loading–unloading up to a strain of 0.8% in a loading rate of 0.05 mm s^−1^. The loading history of one of the ribbons is plotted in Figure S6a (Supporting Information). It was found that the measured moduli of all six specimens were similar, which confirmed that this commercial PVDF sheet is at least transversely isotropic. The *C*_1_ was obtained to be 3.60 ± 0.12 GPa from the slope of this curve.

To characterize the dielectric constant of the commercial PVDF sheet, an LCR meter was used (3532‐50 LCR HiTESTER, HIOKI) and the capacitance of a 1 cm × 1 cm PVDF square was measured to be 324 pF. Hence the capacitance per area is 324 pF cm^−2^. According to the parallel‐plate capacitance equation *C* = *εA*/*h*, where *h* = 28 µm is the thickness of the PVDF, the out‐of‐plane permittivity (ε_3_) of PVDF film could be calculated to be 90.3 pF m^−1^.

*Stiffness and Stretchability of FS PVDF Sensor*: The effective modulus of the FS PVDF sensor and EMAC sensing tattoo is obtainable by performing a uniaxial tensile test on the tattoo, which turned out to be 8.5 MPa according to the stress–strain curve given in Figure 2c. The overall stretchability of the EMAC sensing tattoo can be determined from Figure S9 (Supporting Information), which shows that the FS Au/PET ruptured at 100% whereas the FS PVDF ruptured beyond 112.9%. Such stretchability of the FS network comes from the tortuous serpentine design with nonzero joint angles.

*Electromechanical Behavior of PVDF Ribbons*: The setup to measure the electromechanical behavior of a straight PVDF ribbon and a FS PVDF strain sensor is described in Figure 2a. The output voltage across the thickness of the samples was measured during the mechanical deformation. Each sample was prestretched by a strain of 0.3% and oscillated with an amplitude of 0.2% at a frequency of 1 Hz for 30 s. The experiment was repeated three times for each sample. Due to the high output impedance of PVDF, a voltage follower consisting of an operational amplifier (AD548, Analog Devices) with a high input impedance was used. When calculating the output voltage, the clamped parts of the PVDF sample should be accounted for because *V*_m_ = *Q*_a_/(*C*_a_ + *C*_c_), where *V*_m_ is the measured voltage, *Q*_a_ is the charge generated from the freestanding region, and *C*_a_ and *C*_c_ are the capacitance of the active (red colored in Figure S6d of the Supporting Information) and the clamped (gray colored in Figure S6d of the Supporting Information) parts, respectively. To obtain the genuine output voltage from the active region, *V*_a_, a compensation factor *f* = (*C*_a_ + *C*_c_)/*C*_a_ should be multiplied to *V*_m_, i.e., *V*_a_ = *V*_m_(*C*_a_ + *C*_c_)/*C*_a_. As the thickness and the relative permittivity of the active and clamped PVDF parts are identical (i.e., neglecting the clamping force induced thickness change), *f* can be simplified as *f* = (*A*_a_ + *A*_c_)/*A*_a_. From the given geometry of the straight PVDF ribbon and the FS PVDF strain sensor, we calculated the compensation factors for each sample to be *f*_ST_ = 2.49 and *f*_FS_ = 1.15.

The piezoelectric coefficients (*d*_31,_ *d*_32_) were measured by cyclic uniaxial tests. Two sets of straight PVDF ribbons cut in orthogonal directions were prepared to validate the orthotropy of PVDF electromechanical properties. The piezoelectric coefficient can be obtained using the following equation

(3) $$d_{3j}\approx \frac{\varepsilon_{3}V}{C_{j}hs_{j}}$$

where *V* is the output voltage across the thickness of the PVDF corresponding to the applied strain *s*. Using an applied strain of ±0.2%, the output voltage was measured by a DAQ board (NI‐6225, National Instruments) via a voltage follower circuit (Figure S6c, Supporting Information) and found to be 44.46 V after proper compensation for the clamped region (Figure S6d, Supporting Information). Thus, *d*_31_ was computed to be 10 pC N^−1^ according to Equation (3), which is very different from the value listed in the datasheet (23 pC N^−1^).^17^ In the same way, *d*_32_ was found to be about 1 pC N^−1^, which is 1/10 of *d*_31_. PVDF properties reported by the datasheet and measured ourselves are compared in Table S1 (Supporting Information). Instead of using the properties reported by the datasheet, we used measured PVDF properties as the inputs in all of our FEM analyses.

*FEM of the PVDF Electromechanical Behavior*: Commercial FEM software ABAQUS v6.14 to simulate the output voltage, electric displacement, and mechanical deformation of the FS PVDF strain sensor under uniaxial stretching was used. The FEM models had the same geometries and measured electromechanical properties as described previously. The FS PVDF model was sandwiched between two 47 µm thick Tegaderm layers with widths of 8 mm. Perfect bonding between PVDF and Tegaderm in FEM was assumed. The Young's modulus of Tegaderm was set to be 7 MPa according to the previous measurements.^10^ Poisson's ratios for PVDF and Tegaderm were 0.34 and 0.49, respectively. The electric potential of one metalized surface was set to be 0 (grounded), while that of the other metalized surface was set to be an unknown constant. Regarding the mechanical boundary conditions, one end of the FS PVDF was clamped whereas the other end was uniformly pulled up to 2%. The simulated output voltages are plotted as black dots in Figure 2f. No buckling was observed within the range of the applied strains because Tegaderm suppressed the out‐of‐plane displacement.

FEM for skin‐mounted FS PVDF vibration sensors with different substrates to reveal the substrate effect was also performed. The displacement data at S1 and S2 acquired from the 3D digital image correlation (3D DIC) method was averaged and applied as boundary conditions in the FEM. In this case, the skin is assumed to be a bilayer substrate with 175 µm thick epidermis of 100 kPa modulus and 1.825 mm thick dermis of 20 kPa modulus. The FS PVDF was attached on the epidermis. In one model, there was no substrate covering the FS PVDF. In the other two models the FS PVDF were covered by Tegaderm (7 MPa) and PET (2.5 GPa). Twelve grid points spaced 4.79 mm were assigned on the surface of the epidermis, where 4.79 mm is the spatial resolution of the 3D DIC. The DIC measured displacement data was fitted to be a polynomial function and applied at the surface of the epidermis as boundary conditions. The electrical boundary condition was the same as aforementioned straight PVDF ribbons.

*3D DIC for SCG Measurement*: A subject was properly located in front of two high‐speed cameras (Phantom Miro 310, Vision Research). The distance between the subject and the cameras was ≈55 cm, and the angle between the cameras was 30°. Random black speckles were painted on the surface of the chest by a metal pet brush (Figure S2a, Supporting Information). The subject wore sunglasses to avoid any ocular damage from the LED panel light, which was used to increase the contrast of the target surface and thus improve the quality of the DIC measurement. Before recording, the spatial configuration was calibrated using a two‐level calibration plate (Figure S2b, Supporting Information). The captured images were processed by software (StrainMaster, LaVision) to extract 3D displacement and in‐plane principal strain fields of the chest. The displacement vector and the in‐plane principal strain value were mapped into a 2D array of coordinate points within the area of 200 mm × 180 mm, where the distance between each coordinate point was 4.79 mm.

*Data Acquisition and Signal Processing*: The experimental measurements involved the synchronous measurements of ECG and SCG by the EMAC sensing tattoo, an independent measurement of SCG by a commercial accelerometer (ADXL335, Analog Devices), and an independent continuous BP estimation by a commercial SOMNOtouch NIBP. The SOMNO estimates beat‐to‐beat BP through the PTT method, which requires three gel electrodes on the chest for one channel ECG and one rigid photoplethysmogram (PPG) sensor clipped on the index finger. Before each use, the SOMNO was calibrated using an FDA approved commercial sphygmomanometer (EW3153 Upper Arm Blood Pressure Monitor, Panasonic). Synchronization among different DAQ systems was ensured by aligning the starting time recorded by each device. A diagram for EMAC sensing and BP estimation is displayed in Figure S15a (Supporting Information). First, raw ECG signals sensed by the Au/PET electrodes on the tattoo were passed through the instrumentation amplifier (AD627A, Analog Devices), which is widely used for its advantages of large common mode noise reduction and high input impedance (Figure S15b, Supporting Information). The ECG and SCG signals were both acquired by a multichannel NI‐6225 DAQ box. After collecting 5 beats of ECG and SCG, the signals were filtered by 4th order Butterworth band pass filters with the range of 2–40 Hz for ECG and 12–40 Hz for SCG, respectively (Figure S15c, Supporting Information). Raw and processed ECG and SCG signals from the EMAC tattoo and the accelerometer are plotted in Figure S16 (Supporting Information). To calculate the RAC intervals out of filtered ECG and SCG signals, R peaks from ECG signals were first collected because of their uniqueness. Based on the time of an arbitrary R peak, next R peak within 0.27 s (calculated from the maximum possible human heart rate, 220 bpm) were neglected.^52^ For each detected R peak, the corresponding S1 peak was excluded and only the S2 (i.e., AC) peak was picked up from a period of SCG. Subsequently, beat‐to‐beat RAC intervals were attainable, as described in Figure S17 (Supporting Information). For accurate measurement, mean values of RAC intervals over 5 heartbeats were calculated (Figure S18, Supporting Information) using a moving window.

*Experiments on Human Subjects*: Experiments on human subjects were conducted with the approval from the Institutional Review Board of the University of Texas at Austin (Study number: 2015‐05‐0024), and the consents of all subjects.

## Conflict of Interest

The authors declare no conflict of interest.

## Supporting information

Supplementary

## References

[1] A. Alwan , Global Status Report on Noncommunicable Diseases 2010, World Health Organization, 2011.

[2] a) M. M. Baig , H. Gholamhosseini , M. J. Connolly , Med. Biol. Eng. Comput. 2013, 51, 485; 23334714 10.1007/s11517-012-1021-6

[3] P. K. Jain , A. K. Tiwari , Comput. Biol. Med. 2014, 54, 1. 25194717 10.1016/j.compbiomed.2014.08.014

[4] R. Rhoades , D. R. Bell , Medical Physiology: Principles for Clinical Medicine, Lippincott Williams & Wilkins, Philadelphia 2009.

[5] O. Postolache , P. Girão , G. Postolache , Advanced Instrument Engineering: Measurement, Calibration, and Design: Measurement, Calibration, and Design, IGI Global, Hershey, PA 2013, p. 223.

[6] O. T. Inan , P. F. Migeotte , K. S. Park , M. Etemadi , K. Tavakolian , R. Casanella , J. Zanetti , J. Tank , I. Funtova , G. K. Prisk , M. Di Rienzo , IEEE J. Biomed. Health Inf. 2015, 19, 1414. 10.1109/JBHI.2014.2361732 25312966

[7] Y. Liu , J. J. Norton , R. Qazi , Z. Zou , K. R. Ammann , H. Liu , L. Yan , P. L. Tran , K.‐I. Jang , J. W. Lee , Sci. Adv. 2016, 2, e1601185. 28138529 10.1126/sciadv.1601185 PMC5262452

[8] P. Castiglioni , A. Faini , G. Parati , M. Di Rienzo , in Proc. of the Annual Int. IEEE EMBS, IEEE, Lyon, France 2007, p. 3954. 10.1109/IEMBS.2007.4353199 18002865

[9] D.‐H. Kim , N. Lu , R. Ma , Y.‐S. Kim , R.‐H. Kim , S. Wang , J. Wu , S. M. Won , H. Tao , A. Islam , K. J. Yu , T.‐i. Kim , R. Chowdhury , M. Ying , L. Xu , M. Li , H.‐J. Chung , H. Keum , M. McCormick , P. Liu , Y.‐W. Zhang , F. G. Omenetto , Y. Huang , T. Coleman , J. A. Rogers , Science 2011, 333, 838. 21836009 10.1126/science.1206157

[10] S. X. Yang , Y. C. Chen , L. Nicolini , P. Pasupathy , J. Sacks , B. Su , R. Yang , D. Sanchez , Y. F. Chang , P. L. Wang , D. Schnyer , D. Neikirk , N. S. Lu , Adv. Mater. 2015, 27, 6423. 26398335 10.1002/adma.201502386

[11] S. Kabiri Ameri , R. Ho , H. Jang , L. Tao , Y. Wang , L. Wang , D. M. Schnyer , D. Akinwande , N. Lu , ACS Nano 2017, 11, 7634. 28719739 10.1021/acsnano.7b02182

[12] a) I. Chopra , J. Sirohi , Smart Structures Theory, Vol. 35, Cambridge University Press, New York, NY 2013;

[13] S. Noh , C. Yoon , E. Hyun , H. N. Yoon , T. J. Chung , K. S. Park , H. C. Kim , Electron. Lett. 2014, 50, 143.

[14] S. R. Anton , K. M. Farinholt , A. Erturk , J. Intell. Mater. Syst. Struct. 2014, 25, 1681.

[15] F. M. Hendriks , Technische Universiteit Eindhoven, Eindhoven, Netherlands 2005, p. 106.

[16] a) Y. Qi , J. Kim , T. D. Nguyen , B. Lisko , P. K. Purohit , M. C. McAlpine , Nano Lett. 2011, 11, 1331; 21322604 10.1021/nl104412b

[17] M. Specialties , Measurement 2013, p. 3.

[18] G. Laroche , Y. Marois , R. Guidoin , M. W. King , L. Martin , T. How , Y. Douville , J. Biomed. Mater. Res. 1995, 29, 1525. 8600143 10.1002/jbm.820291209

[19] J. H. Lee , K. Y. Lee , M. K. Gupta , T. Y. Kim , D. Y. Lee , J. Oh , C. Ryu , W. J. Yoo , C. Y. Kang , S. J. Yoon , J. B. Yoo , S. W. Kim , Adv. Mater. 2014, 26, 765. 24167082 10.1002/adma.201303570

[20] W. Y. L. Lionel , G. K. Fedder , IEEE Eng. Med. Biol. 2016, 38, 4816. 10.1109/EMBC.2016.7591805 28269348

[21] W. Dong , L. Xiao , W. Hu , C. Zhu , Y. Huang , Z. Yin , Trans. Inst. Meas. Control 2017, 39, 398.

[22] a) L. Persano , C. Dagdeviren , Y. W. Su , Y. H. Zhang , S. Girardo , D. Pisignano , Y. G. Huang , J. A. Rogers , Nat. Commun. 2013, 4, 1633; 23535654 10.1038/ncomms2639

[23] a) D. H. Kim , R. Ghaffari , N. S. Lu , J. A. Rogers , Annu. Rev. Biomed. Eng. 2012, 14, 113; 22524391 10.1146/annurev-bioeng-071811-150018

[24] S. Liu , T. Ha , N. S. Lu , J. Appl. Mech. 2019, 86, 051010.

[25] a) S. K. Ameri , M. Kim , I. A. Kuang , W. K. Perera , M. Alshiekh , H. Jeong , U. Topcu , D. Akinwande , N. Lu , npj 2D Mater. Appl. 2018, 2, 19;

[26] M. Paukkunen , M. Linnavuo , R. Sepponen , J. Bioeng. Biomed. Sci. 2013, 3, 1.

[27] a) K. Pandia , O. T. Inan , G. T. Kovacs , L. Giovangrandi , Physiol. Meas. 2012, 33, 1643; 22986375 10.1088/0967-3334/33/10/1643

[28] W.‐Y. Lin , W.‐C. Chou , P.‐C. Chang , C.‐C. Chou , M.‐S. Wen , M.‐Y. Ho , W.‐C. Lee , M.‐J. Hsieh , T.‐H. Tsai , M.‐Y. Lee , IEEE J. Biomed. Health. Informs. 2018, 22.2, 442. 10.1109/JBHI.2016.2620496 28113792

[29] a) G. Shafiq , K. C. Veluvolu , Sci. Rep. 2015, 4, 5093; 10.1038/srep05093 PMC4035586 24865183

[30] a) T. Cheng , C. Dai , R. Z. Gan , Ann. Biomed. Eng. 2007, 35, 305; 17160465 10.1007/s10439-006-9227-0

[31] a) S. Omkar , A. Singh , ICTACT J. Image Video Process. 2012, 02, 343;

[32] J. G. Webster , The Physiological Measurement Handbook., CRC Press, 2014, p. 106.

[33] W. Korzeniowska‐Kubacka , M. Bilinska , R. Piotrowicz , Ann. Noninvasive Electrocardiol. 2005, 10, 281. 16029378 10.1111/j.1542-474X.2005.00547.x PMC6932138

[34] J. Sirohi , I. Chopra , J. Intell. Mater. Syst. Struct. 2000, 11, 246.

[35] N. Lu , X. Wang , Z. Suo , J. Vlassak , Appl. Phys. Lett. 2007, 91, 221909.

[36] Y. Hara , Y. Masuda , T. Hirao , N. Yoshikawa , Skin Res. Technol. 2013, 19, 339. 23551131 10.1111/srt.12054

[37] a) J. Y. Sun , N. S. Lu , J. Yoon , K. H. Oh , Z. G. Suo , J. J. Vlassak , J. Mater. Res. 2009, 24, 3338;

[38] K. Pandia , S. Ravindran , R. Cole , G. Kovacs , L. Giovangrandi , presented at 2010 IEEE Int. Conf. on Acoustics Speech and Signal Processing (ICASSP), Dallas, TX, March 2010.

[39] C. Yang , N. Tavassolian , IEEE Sens. J. 2016, 16, 5702.

[40] M. Di Rienzo , P. Meriggi , E. Vaini , P. Castiglioni , F. Rizzo , Annual Int. Conf. of the IEEE on Engineering in Medicine and Biology Society (EMBC), Vol. *34*, San Diego, CA, August–September 2012, p. 5050. 10.1109/EMBC.2012.6347128 23367063

[41] DanielChangMD, DestinyQx, xacax, (Ed: Wiggers_Diagram.svg), Creative Commons Attribution‐Share Alike 4.0 International license, Wikimedia, 2012.

[42] I. Korzeniowska‐Kubacka , B. Kumierczyk‐Droszcz , M. Biliska , B. Dobraszkiewicz‐Wasilewska , K. Piotrowicz , Folia Cardiol. 2006, 13, 319.

[43] a) L. Peter , N. Noury , M. Cerny , IRBM 2014, 35, 271;

[44] S. S. Thomas , V. Nathan , C. Zong , K. Soundarapandian , X. Shi , R. Jafari , IEEE J. Biomed. Health Inf. 2016, 20, 1291. 10.1109/JBHI.2015.2458779 26208369

[45] C. H. Wang , X. S. Li , H. J. Hu , L. Zhang , Z. L. Huang , M. Y. Lin , Z. R. Zhang , Z. N. Yin , B. Huang , H. Gong , S. Bhaskaran , Y. Gu , M. Makihata , Y. X. Guo , Y. S. Lei , Y. M. Chen , C. F. Wang , Y. Li , T. J. Zhang , Z. Y. Chen , A. P. Pisano , L. F. Zhang , Q. F. Zhou , S. Xu , Nat. Biomed. Eng. 2018, 2, 687. 30906648 10.1038/s41551-018-0287-x PMC6428206

[46] a) M. S. Imtiaz , R. Shrestha , T. Dhillon , K. A. Yousuf , B. Saeed , A. Dinh , K. Wahid , IEEE Canadian Conf. on Electrical and Computer Engineering (CCECE), Vol. 26, Regina, SK, Canada, May 2013, p. 501;

[47] X. Y. Zhang , E. MacPherson , Y. T. Hang , IEEE Trans. Biomed. Eng. 2008, 55, 1291. 18390320 10.1109/TBME.2007.912422

[48] H. Tang , J. Gao , Y. Park , J. Biomed. Sci. Eng. 2013, 6, 65.

[49] H. Tang , J. H. Zhang , H. M. Chen , A. Mondal , Y. Park , Physiol. Meas. 2017, 38, 289. 28099168 10.1088/1361-6579/aa552a

[50] a) K. Lu , J. W. Clark , F. H. Ghorbel , D. L. Ware , A. Bidani , Am. J. Physiol.: Heart Circ. Physiol. 2001, 281, H2661; 11709436 10.1152/ajpheart.2001.281.6.H2661

[51] D. R. Mcconahay , C. M. Martin , M. D. Cheitlin , Circulation 1972, 45, 592. 5012247 10.1161/01.cir.45.3.592

[52] H. Tanaka , K. D. Monahan , D. R. Seals , J. Am. College Cardiol. 2001, 37, 153. 10.1016/s0735-1097(00)01054-8 11153730

