# Supplementary material for: A Chest‐Laminated Ultrathin and Stretchable E‐Tattoo for the Measurement of Electrocardiogram, Seismocardiogram, and Cardiac Time Intervals
Source: Adv Sci (Weinh). 2019 May 21;6(14):1900290. doi: 10.1002/advs.201900290 (PMC6662084; doi:10.1002/advs.201900290)
Supplement: Supplementary file 1 — Supplementary [file ADVS-6-1900290-s001.pdf]

Copyright WILEY-VCH Verlag GmbH & Co. KGaA, 69469 Weinheim, Germany, 2019.

ADVANCED SCIENCE
Open Access

Supporting Information

for *Adv. Sci.,* DOI: 10.1002/advs.201900290

A Chest-Laminated Ultrathin and Stretchable E-Tattoo for the Measurement of Electrocardiogram, Seismocardiogram, and Cardiac Time Intervals

*Taewoo Ha, Jason Tran, Siyi Liu, Hongwoo Jang, Hyoyoung Jeong, Ruchika Mitbander, Heeyong Huh, Yitao Qiu, Jason Duong, Rebecca L. Wang, Pulin Wang, Animesh Tandon, Jayant Sirohi, and Nanshu Lu**

Supporting Information

A soft, noninvasive e-tattoo for the measurement of electrocardiogram, seismocardiogram, cardiac time intervals and for the estimation of beat-to-beat blood pressure

Taewoo Ha[1], Jason Tran[2], Siyi Liu[2], Hongwoo Jang[3], Hyoyoung Jeong[1], Ruchika Mitbander[4], Heeyong Huh[5], Yitao Qiu[2], Jason Duong[4], Rebecca L. Wang[2], Pulin Wang[2], Animesh Tandon[6], Jayant Sirohi[2], Nanshu Lu[1, 2, 3, 4, 5*]

[1] *Department of Electrical and Computer Engineering, University of Texas at Austin,* [2] *Department of Aerospace Engineering and Engineering Mechanics, University of Texas at Austin,* [3] *Texas Materials Institute, University of Texas at Austin,* [4] *Department of Biomedical Engineering, University of Texas at Austin,* [5] *Department of Mechanical Engineering, University of Texas at Austin,* [6] *Departments of Pediatrics, Radiology, and Biomedical Engineering, Division of Cardiology, University of Texas Southwestern Medical School, Children's Medical Center Dallas.*

* Corresponding author. Tel.:+1 512-471-4208, Email: nanshulu@utexas.edu, Address: 2501 Speedway, EER 7.614, Austin, TX 78712

Supplementary Information

Supplementary Note 1

Supplementary Materials

Supplementary Figure S1-S18

Supplementary Table S1-S3

Supplementary Note S1: Correlation between HR, BP, and RAC

Although HR and BP are sometimes related, their correlation is not generic and can be easily changed. Plotting data collected among the general population in the same chart shows that resting HR is positively proportional to SBP and DBP[1]. For individuals, a report by Polinski and co-authors concluded that the correlation between HR and BP over the course of a few days seems to be random although short-term correlation is observable [2].

Regarding HR and RAC, Castro and colleagues observed a strong positive correlation between HR and RAC which were measured every two minutes in steady states after several continuous workloads [3]. In a similar experiment, Zhang *et al*. found that both HR and BP increased instantly after exercise, but the recovery rate of HR was slower than that of BP during the resting phase while RAC showed a significant negative correlation with BP for most subjects [4]. However, since exercise increases both HR and BP [5], it is difficult to conclude whether BP is more correlated with HR or RAC.

To investigate the BP-HR and RAC-HR correlations, we list their Pearson correlation coefficients (R) obtained by our Valsalva maneuver experiments for comparison in Table S3. We find that even for the individual subject, HR does not show a meaningful correlation to either RAC or BP whereas we already know that RAC and BP exhibit a much stronger correlation as shown in Table 1. Figure S19 plots the RAC, BP and HR measured continuously before and after

two Valsalva maneuvers of two different subjects. For most subjects as represented by the two shown, HR dropped instantly to the baseline or even lower right after the Valsalva maneuver, which is known as the reflex bradycardia. In contrast, BP and RAC demonstrated much more gradual changes, which is agreeable to previous papers [6]. Based on our own results, we conclude that RAC rather than HR is correlated with BP.

References

[1] M. Valentini, G. Parati, Prog Cardiovasc Dis 2009, 52, 11.

[2] A. Poliński, J. Kot, A. Meresta, "Analysis of correlation between heart rate and blood pressure", presented at *Computer Science and Information Systems (FedCSIS), 2011 Federated Conference on*, 2011.

[3] A. Castro, A. Moukadem, S. Schmidt, A. Dieterlen, M. T. Coimbra, "Analysis of the Electromechanical Activity of the Heart from Synchronized ECG and PCG Signals of Subjects Under Stress", presented at *Proceedings of the International Conference on Bio-inspired Systems and Signal Processing, Biosignals 2015, Biostec, 12-15 January 2015, Lisbon, Portugal*, 2015.

[4] X. Y. Zhang, E. MacPherson, Y. T. Hang, Ieee T Bio-Med Eng 2008, 55, 1291.

[5] D. Pramanik, *Principles of physiology*, Academic publishers, 2007; W. E. Clutter, D. M. Bier, S. D. Shah, P. E. Cryer, J Clin Invest 1980, 66, 94.

[6] F. Y. Liang, H. Liu, J Physiol Sci 2006, 56, 45; J. R. Daube, D. I. Rubin, *Clinical neurophysiology*, Oxford University Press, Oxford ; New York 2009.

Supplementary Materials

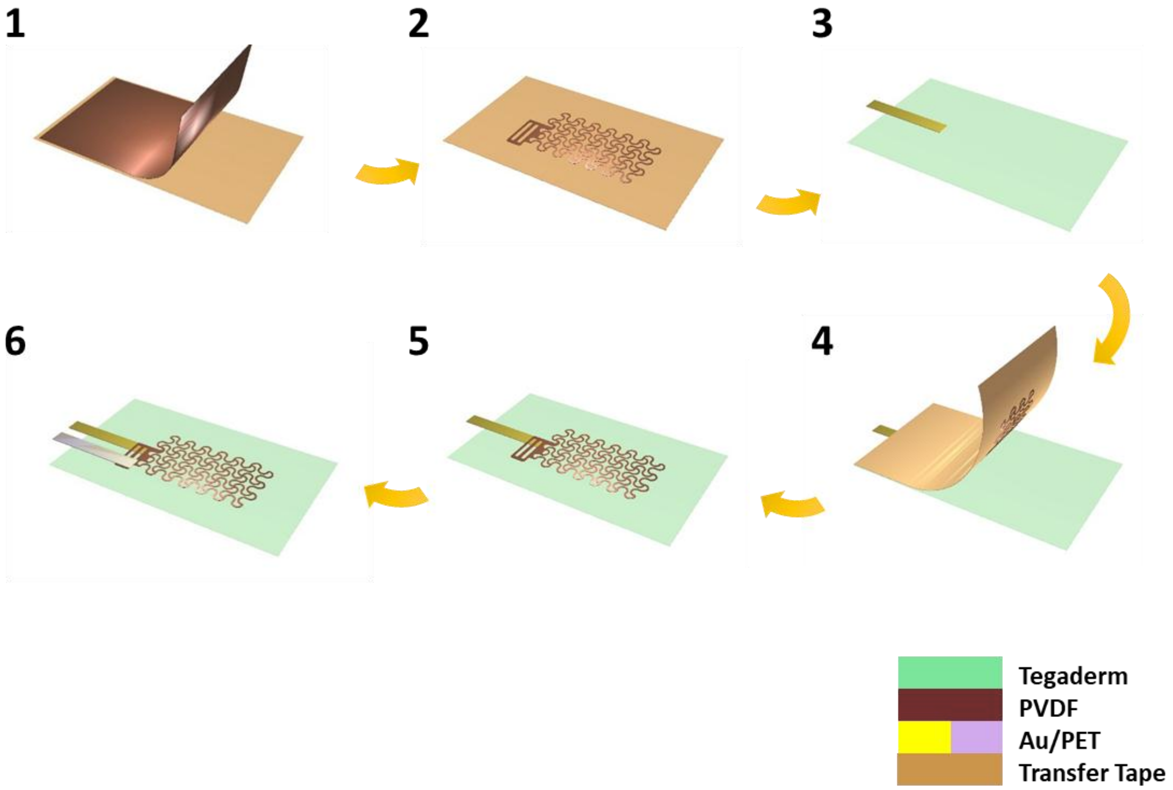

Figure S1. Schematics of the cut-and-paste fabrication process of the FS PVDF strain sensor. After patterning the PVDF film from the transfer tape, the patterned PVDF is transferred to the Tegaderm (1-4). Two electrodes are required to collect charges from the top and bottom electrode of PVDF separately (5-6).

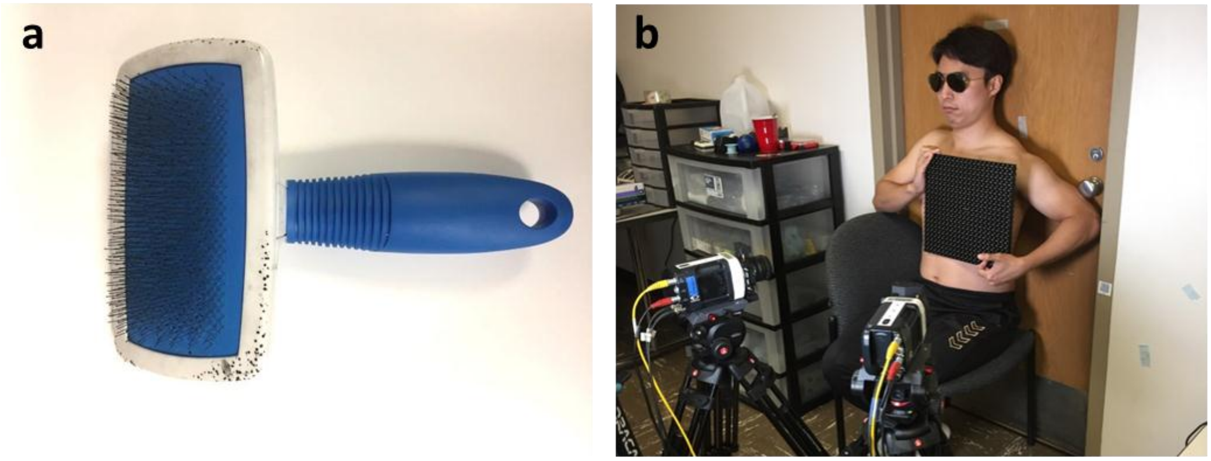

Figure S2. a) A photograph of a pet brush for patterning speckles on the chest. b) A photograph of 3D DIC method. A subject holding a calibration plate.

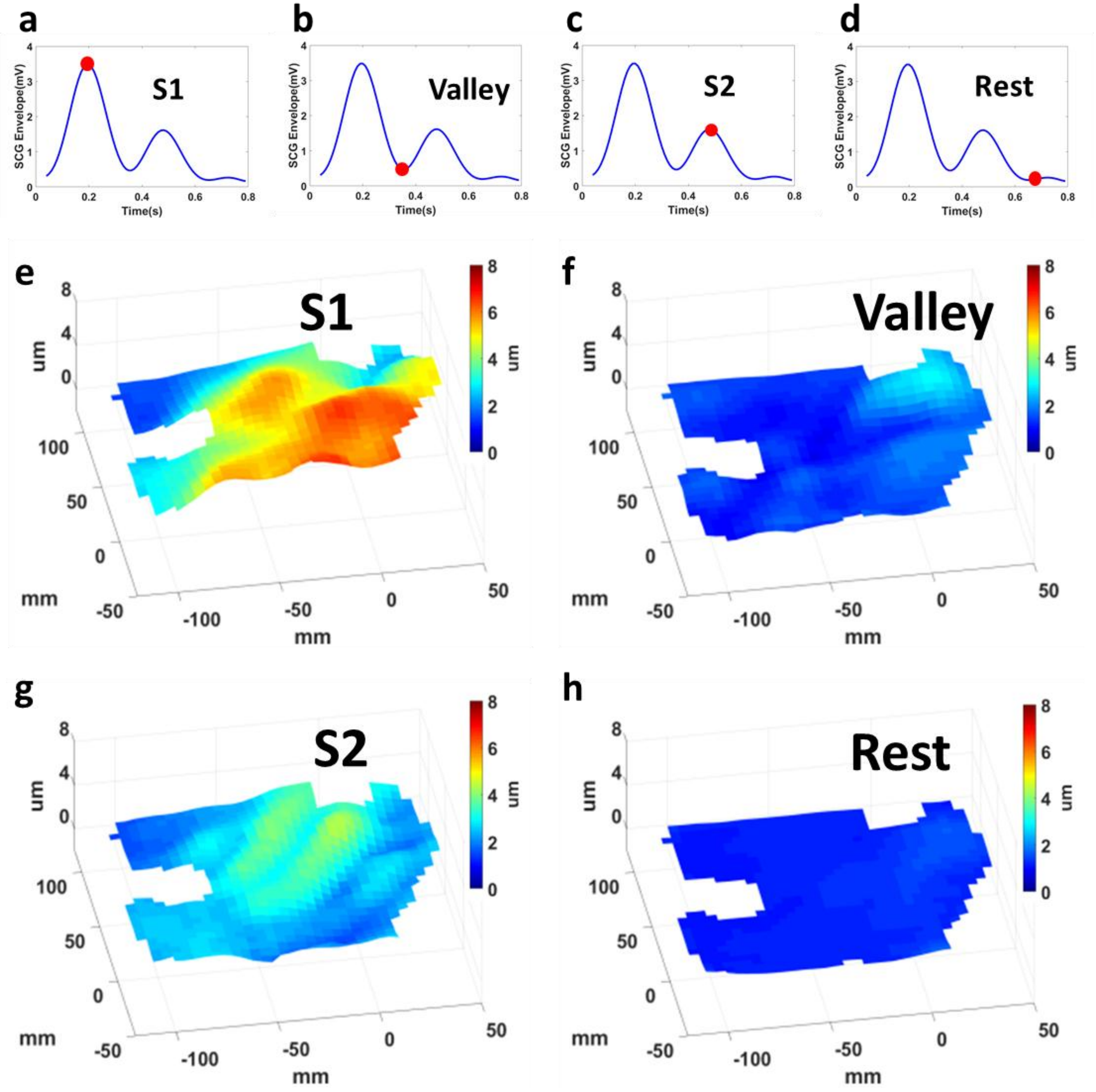

Figure S3. a-d) A periodic cycle of SCG envelope (S1-Valley-S2-Rest) and e-h) corresponding full-field 3D map.

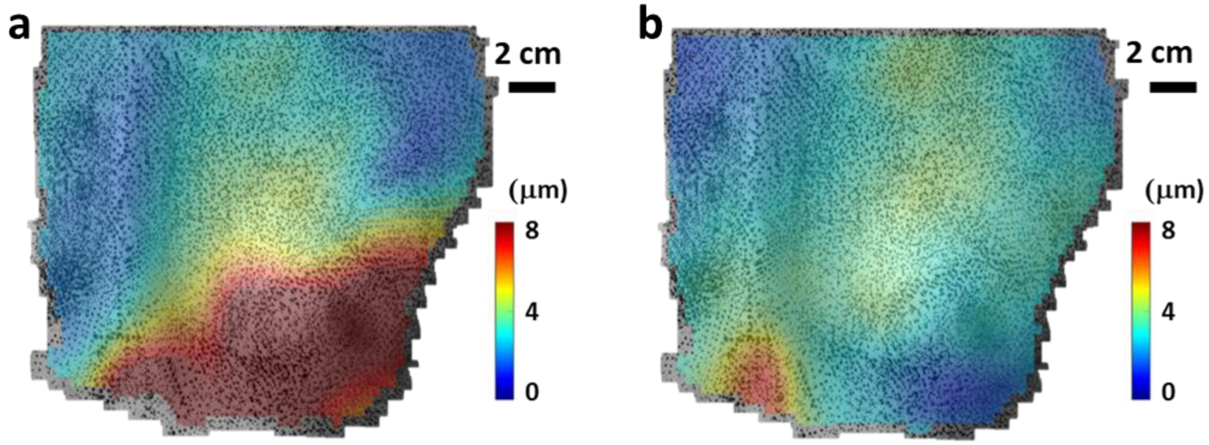

Figure S4. A full-field out-of-plane displacement map at a) S1 and b) S2, derived from Subject 2 using 3D DIC method. Full-field out-of-plane displacement maps at S1/S2 within 16 seconds are averaged.

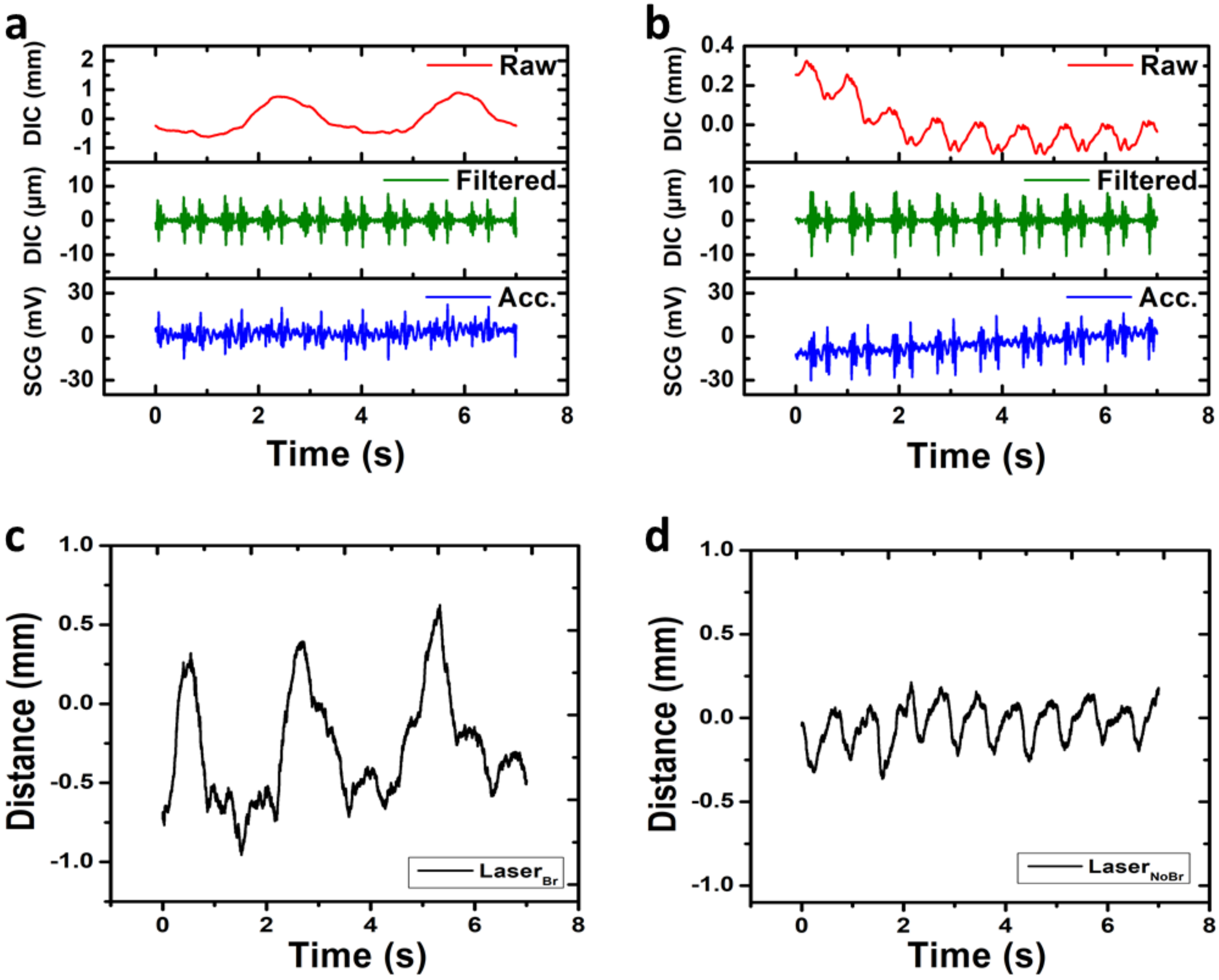

Figure S5. Measured signals by 3D DIC method (Raw, Filtered) and the accelerometer (Acc.) during a) breathing and b) holding breath. Laser displacement measurement during c) breathing and d) holding breath.

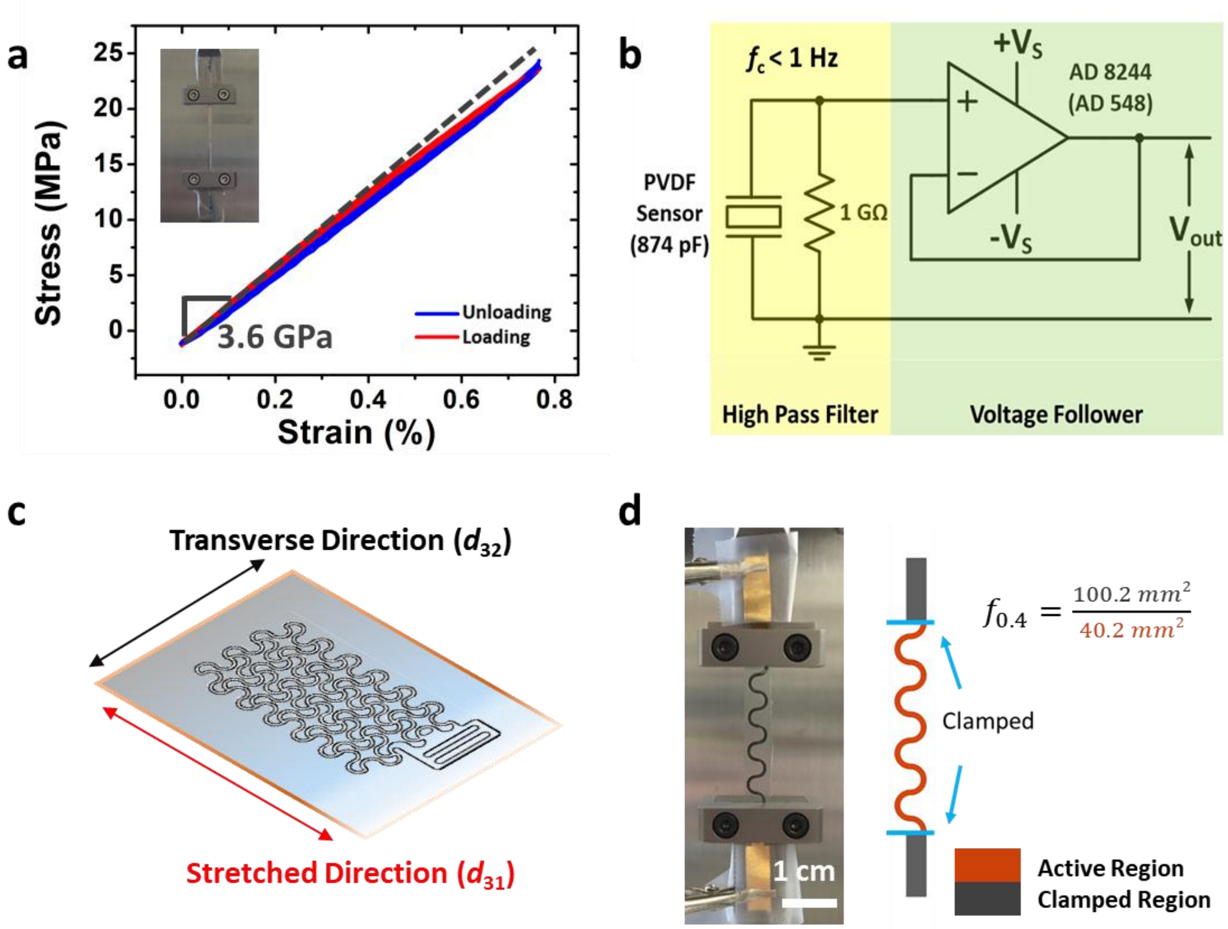

Figure S6. a) A representative stress-strain curve of a straight PVDF ribbon. b) A voltage follower circuit for piezoelectric sensors. c) The pattern of FS PVDF sensor with respect to the stretched direction and the transverse direction of PVDF sheet. d) A photograph of a serpentine-shaped PVDF ribbon under the tensile test and an illustration of the voltage compensation factor *f*.

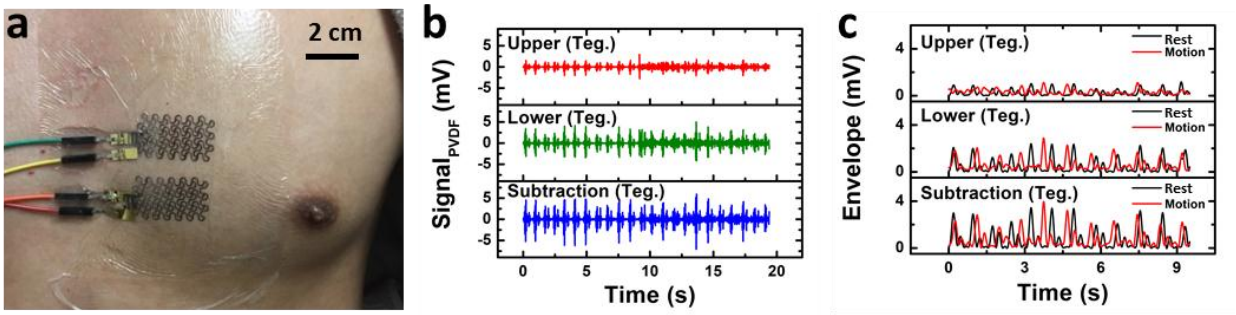

Figure S7. Dual-sensor based motion artifact cancellation with Tegaderm. a) A photograph of the dual FS PVDF sensing system (Tegaderm covered) attached on the chest of a subject. b) Filtered SCG signals and c) corresponding envelope signals recorded by upper and lower FS PVDF strain sensors and their subtraction result under rest/motion condition.

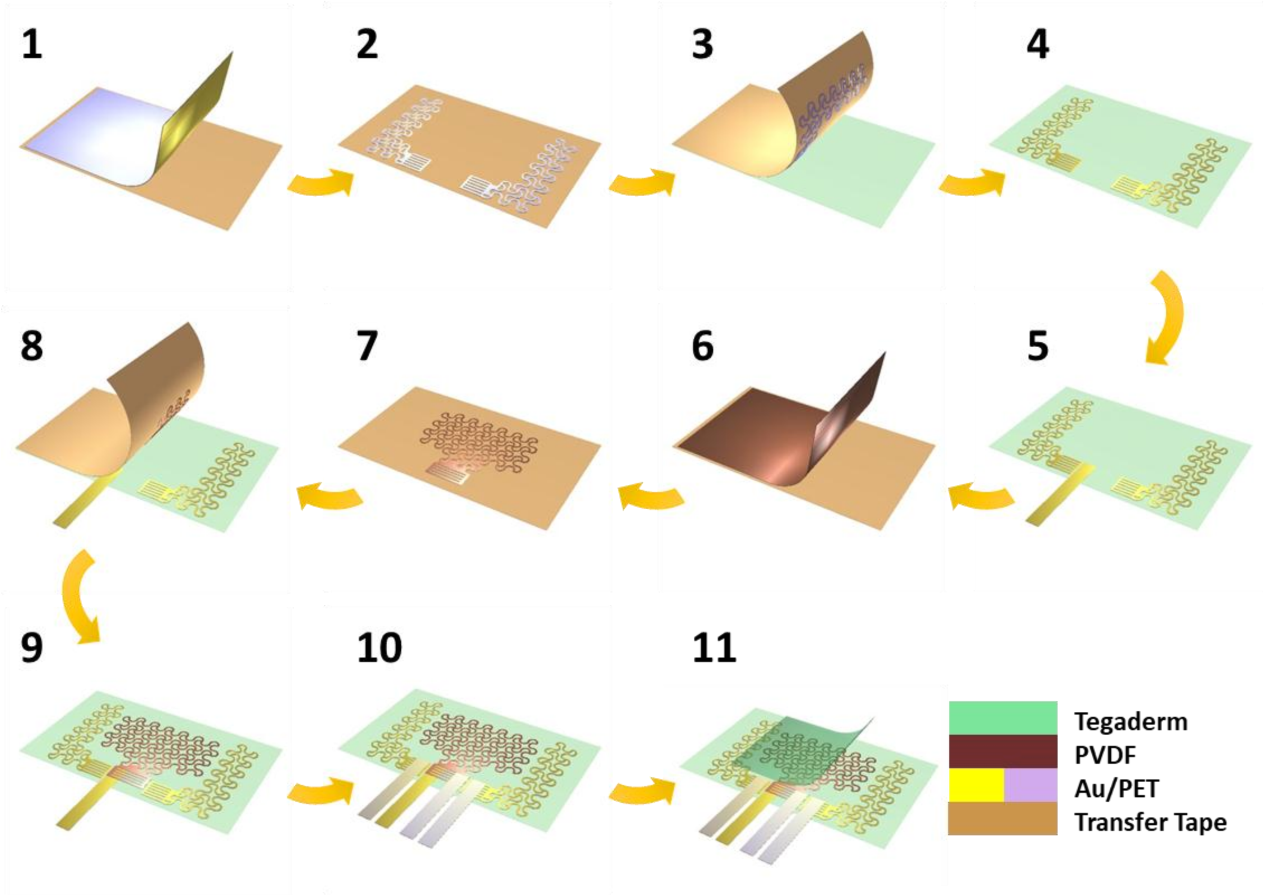

Figure S8. The fabrication process of the EMAC sensing tattoo.

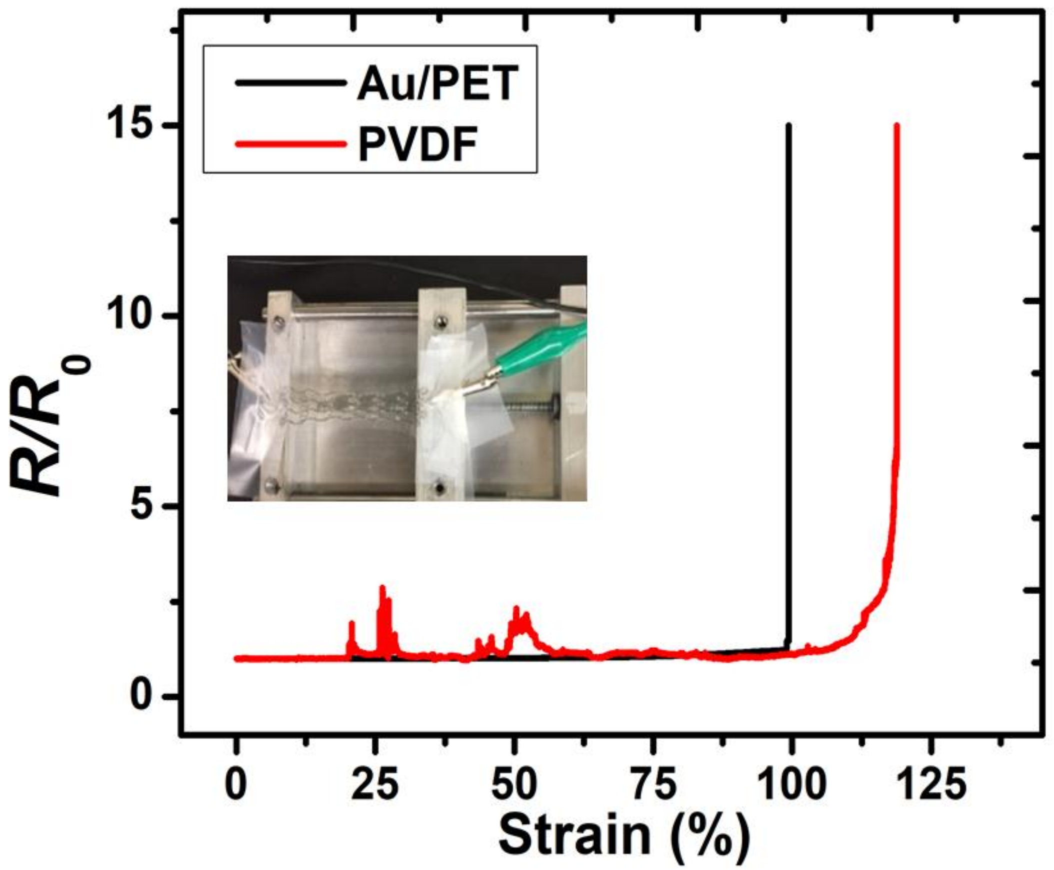

Figure S9. The stretchability of the FS Au/PET and the FS PVDF. The FS Au/PET electrode ruptures before the FS PVDF.

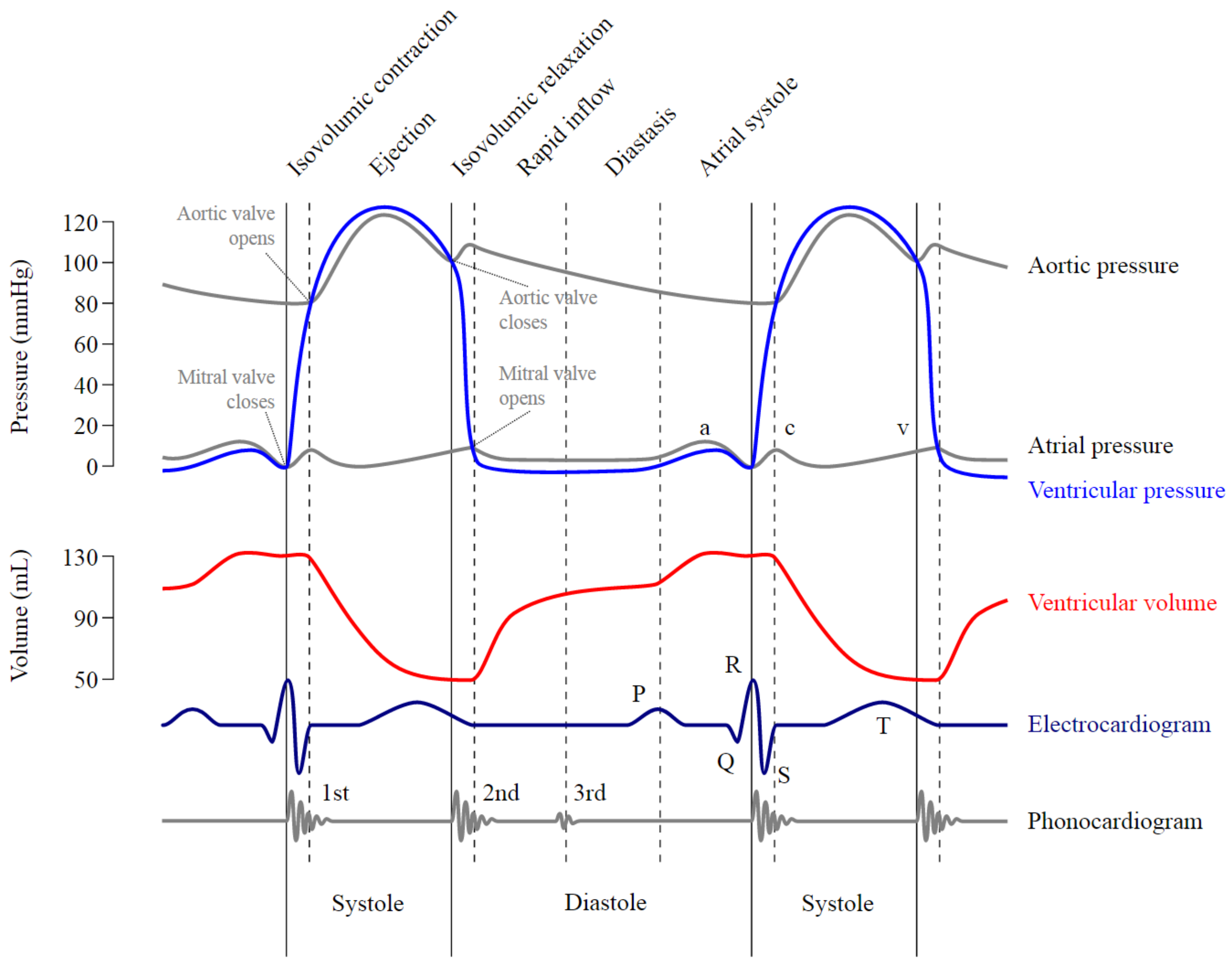

Figure S10. Wigger’s diagram by DanielChangMD who revised original work of DestinyQx; Redrawn as SVG by xavax, 2012, via Wikimedia Commons. Used under a Creative Commons Attribution-Share Alike 4.0 International license.

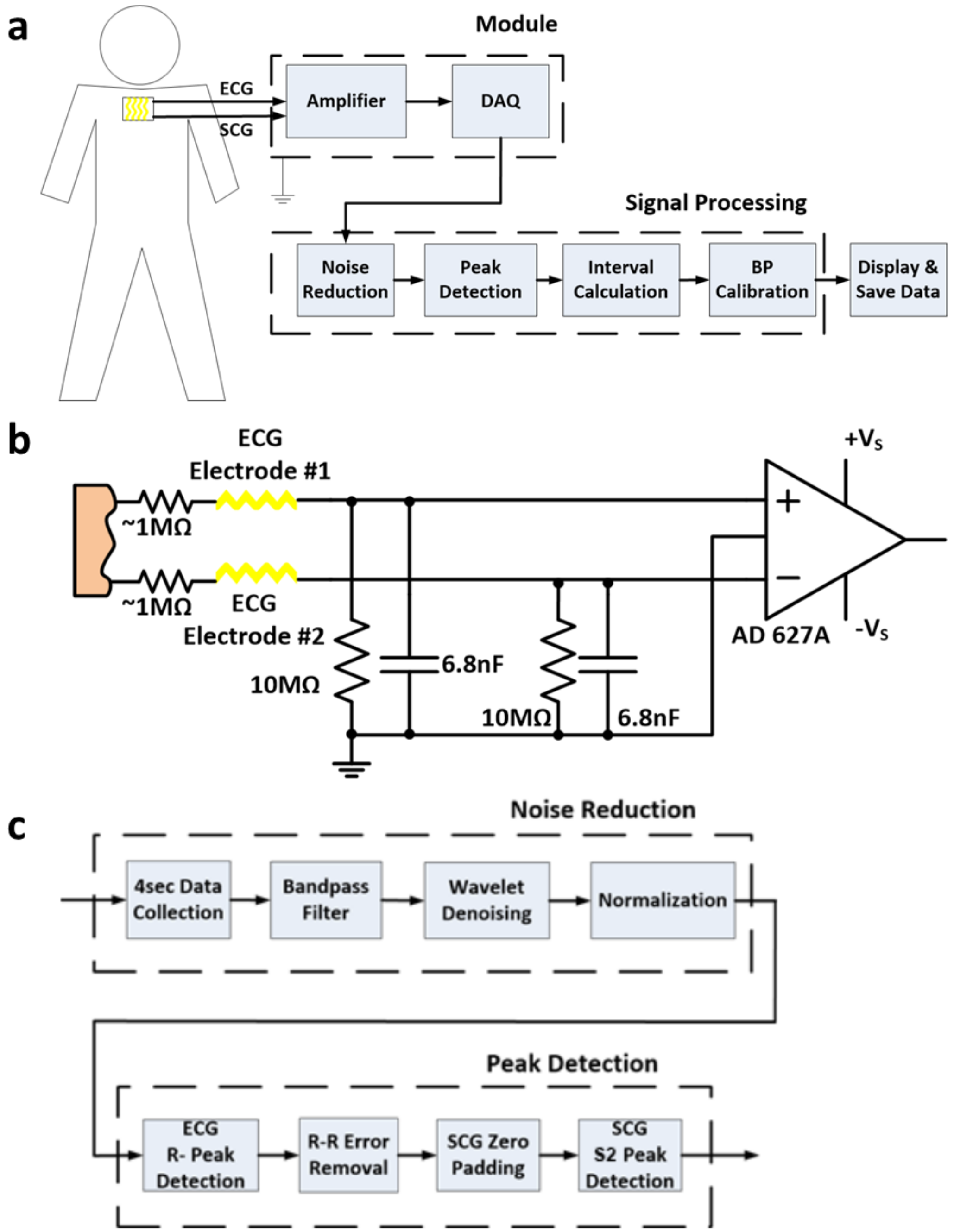

Figure S11. Illustrations of the system for ECG and SCG acquisition and signal processing. a) System flow chart. ECG and SCG are collected by a signal acquisition module from a subject. Then, the collected signals are processed to calculate the interval between R peak of ECG and AC peak of SCG. b) Custom circuits for ECG measurement. c) Noise reduction and peak detection algorithm flow chart.

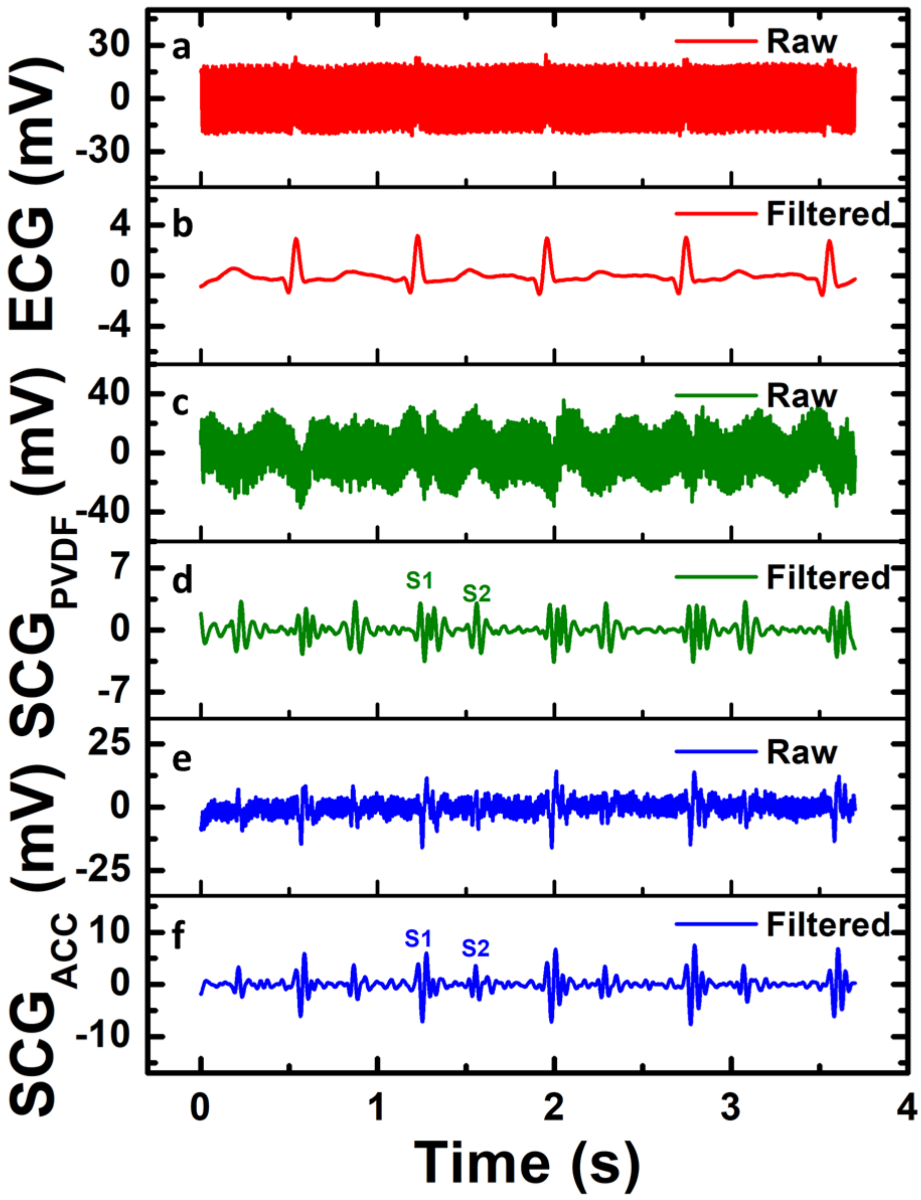

Figure S12. Collected and filtered signals from an EMAC sensing tattoo and an accelerometer on the chest. a) Raw ECG signal measured by a Au/PET sensor. b) ECG Signal filtered with 2-40Hz, 4th order Butterworth bandpass filter. c) Raw SCG measured by a FS PVDF sensor (SCG-PVDF). d) SCG-PVDF signal filtered with 12-40Hz, 4th order Butterworth bandpass filter. e) Raw SCG signal measured by an ADXL-335 accelerometer (SCG-ACC). f) SCG-ACC signal filtered with 12-40Hz, 4th order Butterworth bandpass filter..

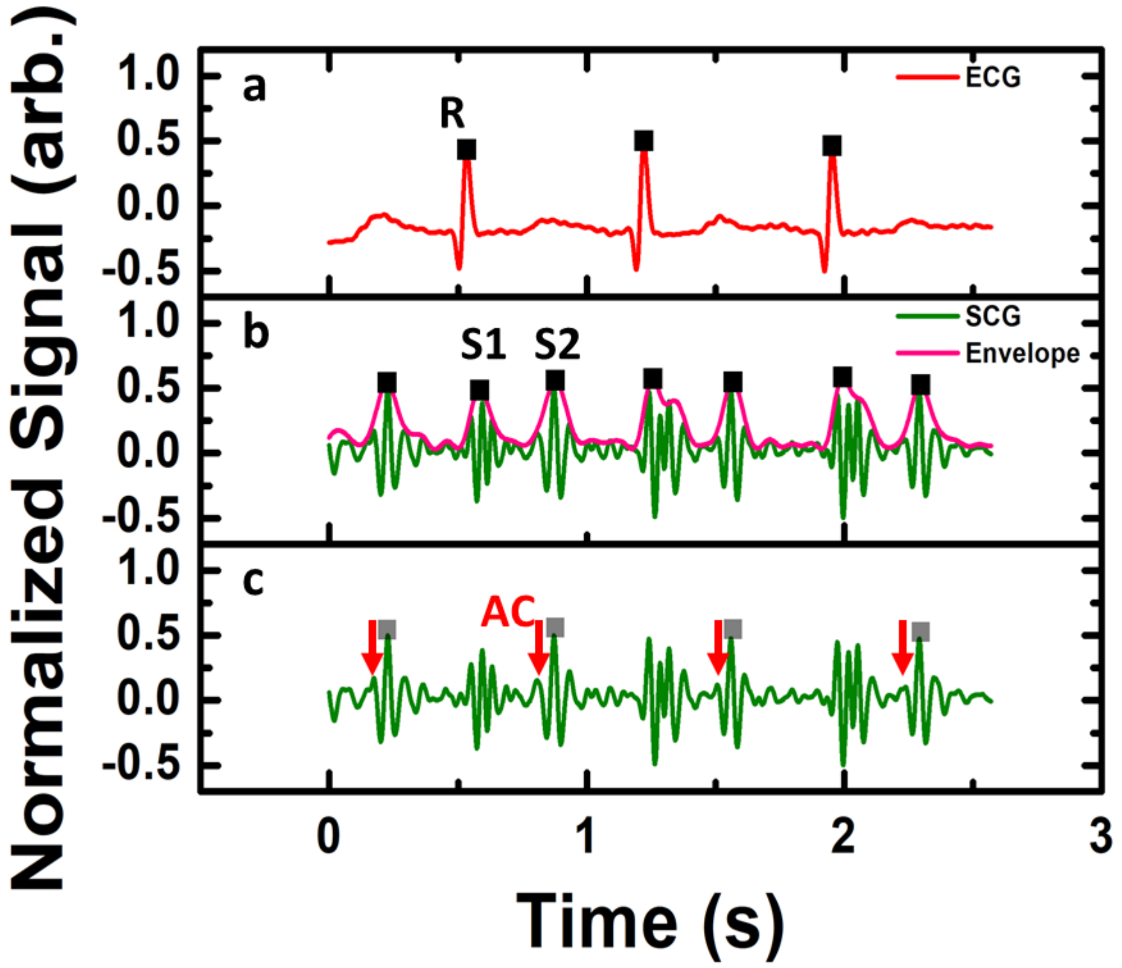

Figure S13. RAC interval detection. a) Normalized ECG signal. Black dots indicate R peaks of ECG. b) Normalized SCG signal. Black dots indicate S1 and S2 peaks of SCG. c) Normalized, S1-padded SCG signal. Gray dots indicate S2 peaks of SCG only. AC peaks can be detected based on the location of S2 peaks.

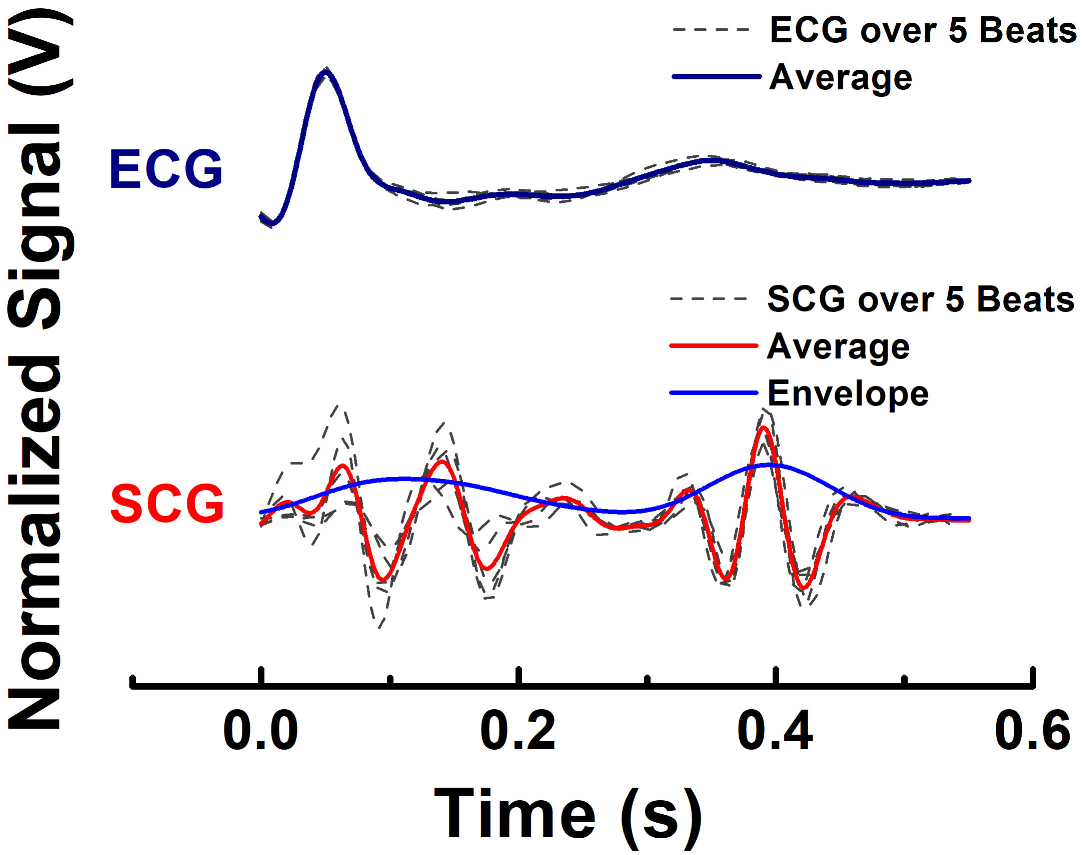

Figure S14. Averaged ECG and SCG signals over 5 heartbeats. Navy color and Red color represent averaged ECG and SCG signals over 5 heartbeats, respectively. Blue colored line represents the envelope of the averaged SCG signal.

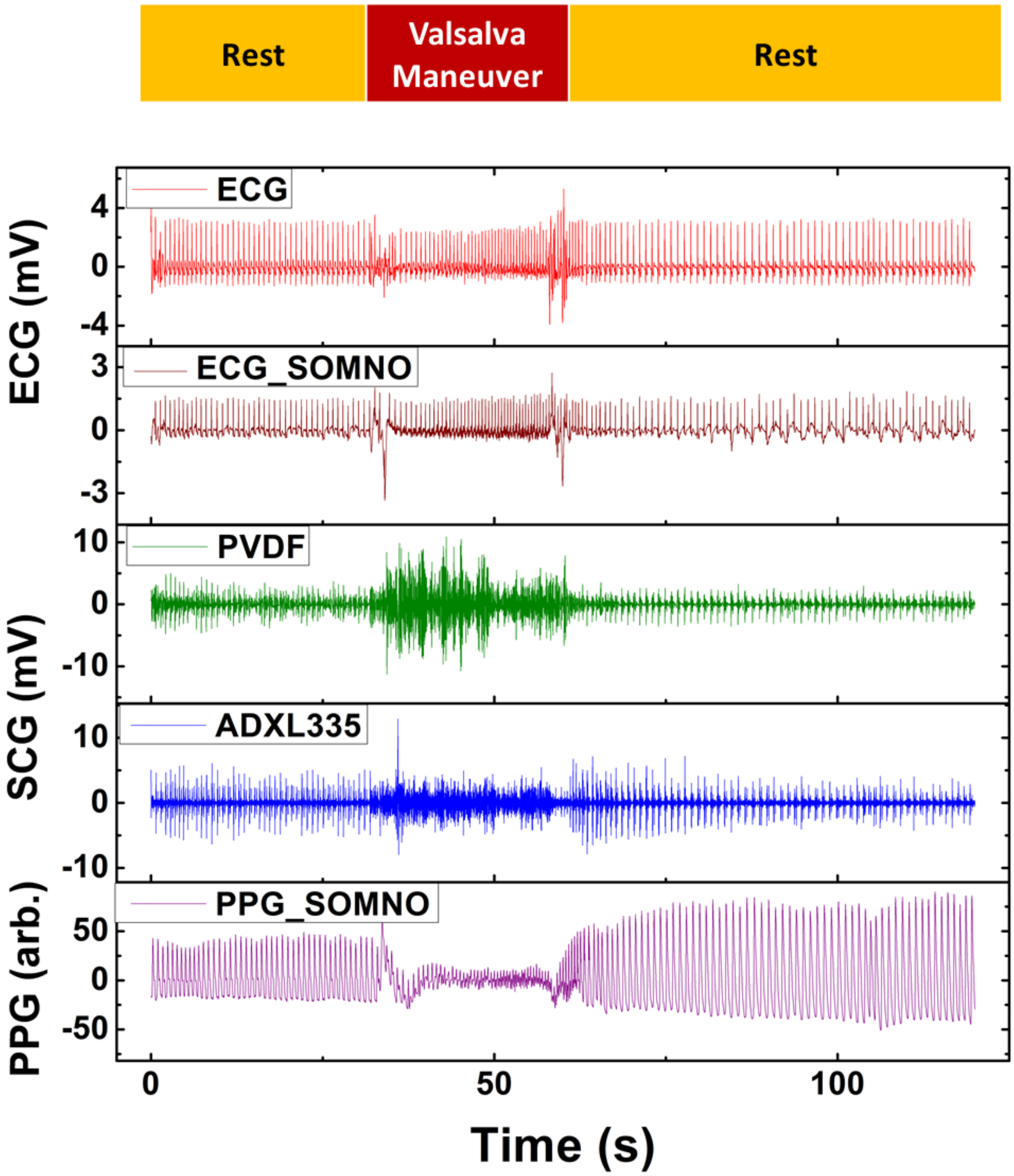

Figure S15. Entire ECG, SCG, and PPG signals collected from both EMAC sensing tattoo and SOMNOtouch^{TM} NIBP during the Valsalva maneuver experiment.

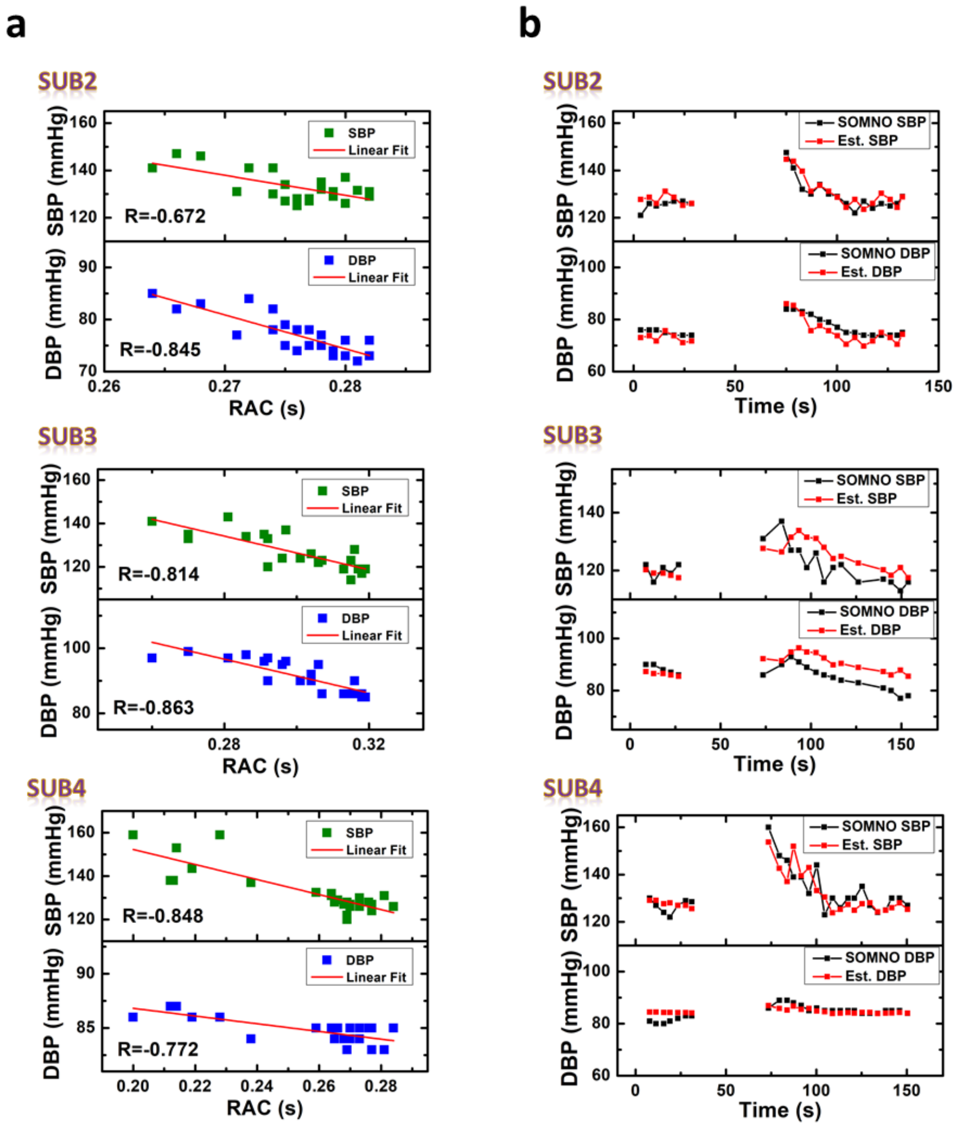

Figure S16. a) SBP/DBP vs RAC correlation chart and its Pearson correlation coefficient (R) from the first experiment of each subject. Red line represents the fitting curve, $y = ax + b$. Fitting parameters of each subject were decided from the chart. R is Pearson's correlation coefficient. b) Comparison between SBP(DBP) measured by SOMNO NIBP and EMAC sensing patch. Estimated SBP/DBP was calculated as a function of the mean RAC of the second experiment with the fitting parameters of the first experiment.

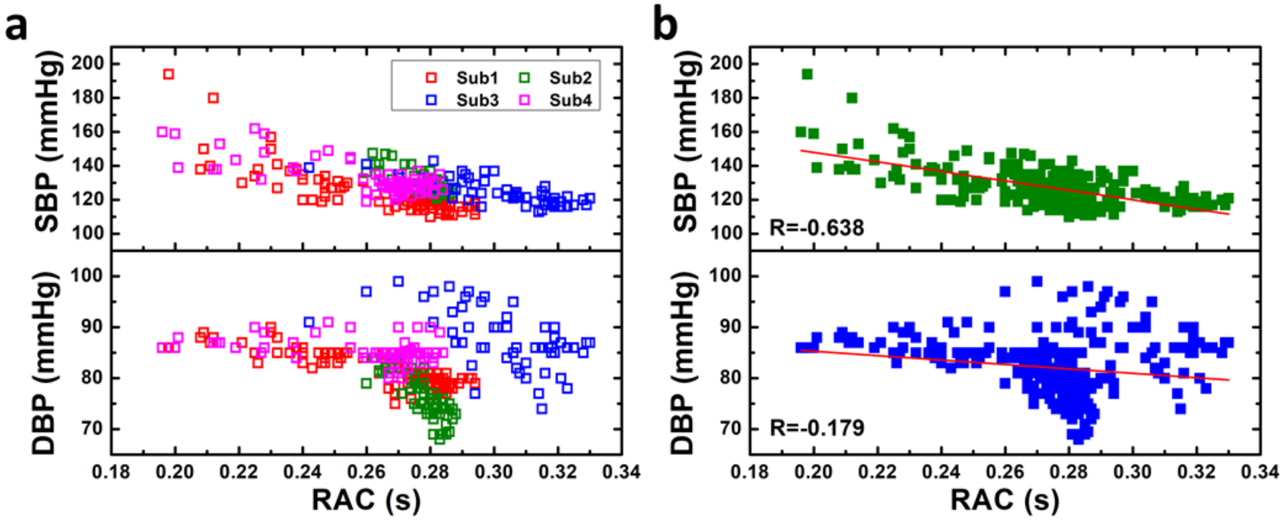

Figure S17. a) SBP/DBP and RAC correlation chart among four different subjects. b) SBP/DBP and RAC correlation of all subjects.

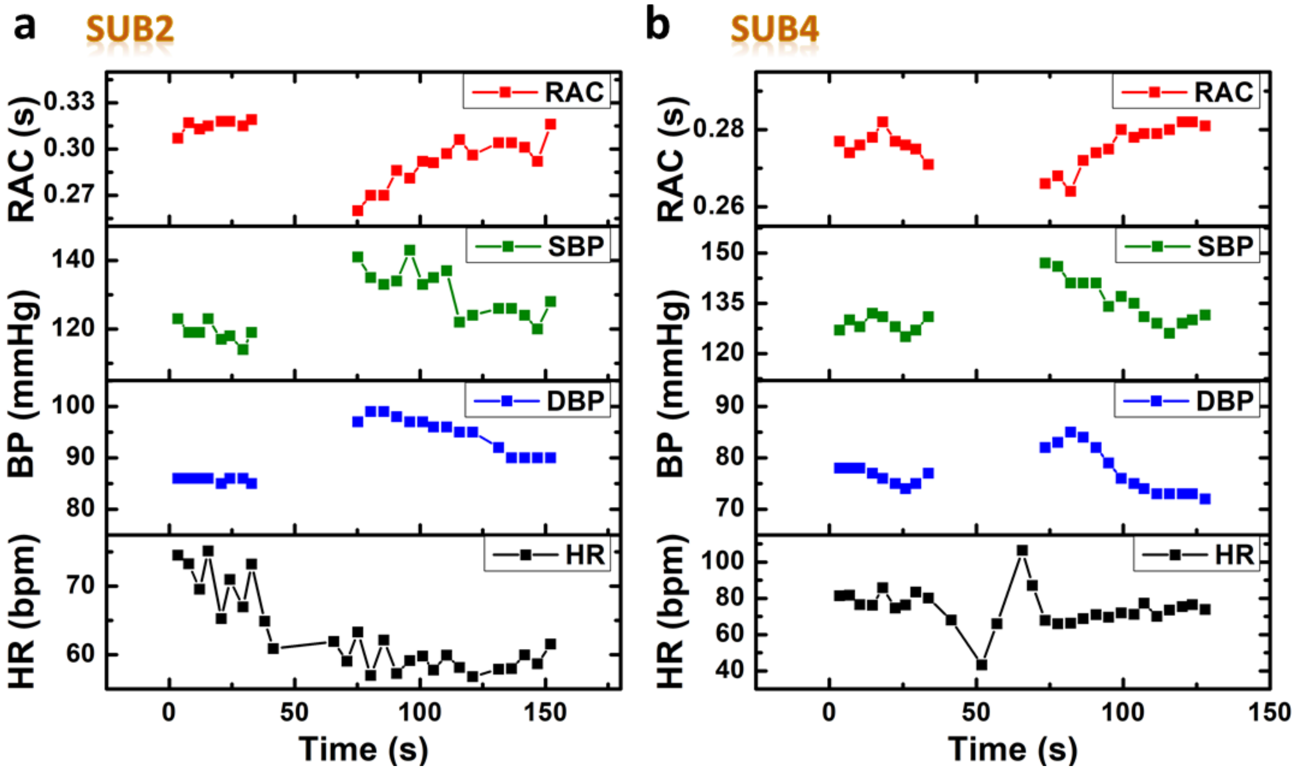

Figure S18 Synchronously measured HR, BP and RAC during the Valsalva maneuver experiment. Some points were omitted because of the motion artifact.

	Datasheet	Measured Value
**In-plane stiffness C_{11} (GPa)**	2 – 4	3.6
**Capacitance per area (pF/cm^2)**	380	324
**Out of plane relative permittivity**	12 – 13	10.2
**Piezo strain constant d_{31} (pC/N)**	23	10
**Voltage per μ-strain (mV)**	12	11.1

Table S1. Comparison between the data sheet and actual measurement of piezofilm.

Subject	HR,RAC	HR,SBP	HR,DBP
**Sub1**	-0.685	0.743	0.853
**Sub2**	0.235	0.240	0.104
**Sub3**	-0.404	0.466	0.322
**Sub4**	-0.328	0.404	0.347
**Average**	-0.296	0.463	0.407

Table S2. Association among HR, BP and RAC based on Pearson's correlation coefficient.

Subject	SBP (mmHg)	DBP (mmHg)	HR (bpm)	Age	Height (cm)	Weight (kg)
**Sub1**	119	80	74	28	177	79
**Sub2**	117	72	70	28	180	75
**Sub3**	127	78	85	34	183	81
**Sub4**	129	74	66	23	185	72
**Average**	123	76	73.75	28.25	181.25	76.75

Table S3. Physical and cardiovascular parameters of subjects at normal state.
